# The fallacy of placing confidence in confidence intervals

**DOI:** 10.3758/s13423-015-0947-8

**Published:** 2015-10-08

**Authors:** Richard D. Morey, Rink Hoekstra, Jeffrey N. Rouder, Michael D. Lee, Eric-Jan Wagenmakers

**Affiliations:** Cardiff University, Cardiff, UK; University of Groningen, Groningen, Netherlands; University of Missouri, Columbia, MO USA; University of California-Irvine, Irvine, CA USA; University of Amsterdam, Amsterdam, Netherlands

**Keywords:** Bayesian inference and parameter estimation, Bayesian statistics, Statistical inference, Statistics

## Abstract

Interval estimates – estimates of parameters that include an allowance for sampling uncertainty – have long been touted as a key component of statistical analyses. There are several kinds of interval estimates, but the most popular are confidence intervals (CIs): intervals that contain the true parameter value in some known proportion of repeated samples, on average. The width of confidence intervals is thought to index the precision of an estimate; CIs are thought to be a guide to which parameter values are plausible or reasonable; and the confidence coefficient of the interval (e.g., 95 %) is thought to index the plausibility that the true parameter is included in the interval. We show in a number of examples that CIs do not necessarily have any of these properties, and can lead to unjustified or arbitrary inferences. For this reason, we caution against relying upon confidence interval theory to justify interval estimates, and suggest that other theories of interval estimation should be used instead.

**Electronic supplementary material** The online version of this article (doi:10.3758/s13423-015-0947-8) contains supplementary material, which is available to authorized users.

“You keep using that word. I do not think it means what you think it means.” Inigo Montoya, *The Princess Bride* (1987)

The development of statistics over the past century has seen the proliferation of methods designed to allow inferences from data. Methods vary widely in their philosophical foundations, the questions they are supposed to address, and their frequency of use in practice. One popular and widely-promoted class of methods comprises interval estimates. There are a variety of approaches to interval estimation, differing in their philosophical foundation and computation, but informally all are supposed to be estimates of a parameter that account for measurement or sampling uncertainty by yielding a range of values for the parameter instead of a single value.

Of the many kinds of interval estimates, the most popular is the confidence interval (CI). Confidence intervals are introduced in almost all introductory statistics texts; they are recommended or required by the methodological guidelines of many prominent journals (e.g., Psychonomics Society, 2012; Wilkinson and the Task Force on Statistical Inference, 1999); and they form the foundation of methodological reformers’ proposed programs (Cumming 2014; Loftus 1996). In the current atmosphere of methodological reform, a firm understanding of what sorts of inferences confidence interval theory does, and does not, allow is critical to decisions about how science is to be done in the future.

In this paper, we argue that advocacy of CIs is based on a folk understanding rather than a principled understanding of CI theory. We outline three fallacies underlying the folk theory of CIs and place these in the philosophical and historical context of CI theory proper. Through an accessible example adapted from the statistical literature, we show how CI theory differs from the folk theory of CIs. Finally, we show the fallacies of confidence in the context of a CI advocated and commonly used for ANOVA and regression analysis, and discuss the implications of the mismatch between CI theory and the folk theory of CIs.

Our main point is this: confidence intervals should not be used as modern proponents suggest because this usage is not justified by confidence interval theory. The benefits that modern proponents see CIs as having are considerations outside of confidence interval theory; hence, if used in the way CI proponents suggest, CIs can provide severely misleading inferences. For many CIs, proponents have not actually explored whether the CI supports reasonable inferences or not. For this reason, we believe that appeal to CI theory is redundant in the best cases, when inferences can be justified outside CI theory, and unwise in the worst cases, when they cannot.

## The folk theory of confidence intervals

In scientific practice, it is frequently desirable to estimate some quantity of interest, and to express uncertainty in this estimate. If our goal were to estimate the true mean *μ* of a normal population, we might choose the sample mean $\bar {x}$ as an estimate. Informally, we expect $\bar {x}$ to be close to *μ*, but *how* close depends on the sample size and the observed variability in the sample. To express uncertainty in the estimate, CIs are often used.

If there is one thing that everyone who writes about confidence intervals agrees on, it is the basic definition: A confidence interval for a parameter — which we generically call *θ* and might represent a population mean, median, variance, probability, or any other unknown quantity — is an interval generated by a procedure that, on repeated sampling, has a fixed probability of containing the parameter. If the probability that the process generates an interval including *θ* is .5, it is a 50 % CI; likewise, the probability is .95 for a 95 % CI.

### **Definition 1** (Confidence interval)

*An X% confidence interval for a parameterθis an interval (L,U) generated by a procedure that in repeated sampling has an X% probability of containing the true value of θ, for all possible values of θ* (Neyman 1937).^1^

The confidence coefficient of a confidence interval derives from the procedure which generated it. It is therefore helpful to differentiate a *procedure* (CP) from a confidence *interval*: an *X*% confidence procedure is any procedure that generates intervals cover *θ* in *X*% of repeated samples, and a confidence interval is a specific interval generated by such a process. A confidence procedure is a random process; a confidence interval is observed and fixed.

It seems clear how to interpret a confidence *procedure*: it is any procedure that generates intervals that will cover the true value in a fixed proportion of samples. However, when we compute a specific interval from the data and must interpret it, we are faced with difficulty. It is not obvious how to move from our knowledge of the properties of the confidence procedure to the interpretation of some observed confidence interval.

Textbook authors and proponents of confidence intervals bridge the gap seamlessly by claiming that confidence intervals have three desirable properties: first, that the confidence coefficient can be read as a measure of the uncertainty one should have that the interval contains the parameter; second, that the CI width is a measure of estimation uncertainty; and third, that the interval contains the “likely” or “reasonable” values for the parameter. These all involve reasoning about the parameter from the observed data: that is, they are “post-data” inferences.

For instance, with respect to 95 % confidence intervals, Masson and Loftus (2003) state that “in the absence of any other information, there is a 95 % probability that the obtained confidence interval includes the population mean.”Cumming (2014) writes that “[w]e can be 95 % confident that our interval includes [the parameter] and can think of the lower and upper limits as likely lower and upper bounds for [the parameter].”

These interpretations of confidence intervals are not correct. We call the mistake these authors have made the “Fundamental Confidence Fallacy” (FCF) because it seems to flow naturally from the definition of the confidence interval:

### **Fallacy 1** (The Fundamental Confidence Fallacy)

*If the probability that a random interval contains the true value is X%, then the plausibility or probability that a particular observed interval contains the true value is also X%; or, alternatively, we can have X% confidence that the observed interval contains the true value.*

The reasoning behind the Fundamental Confidence Fallacy seems plausible: on a given sample, we could get any one of the possible confidence intervals. If 95 % of the possible confidence intervals contain the true value, without any other information it seems reasonable to say that we have 95 % certainty that we obtained one of the confidence intervals that contain the true value. This interpretation is suggested by the name “confidence interval” itself: the word “confident”, in lay use, is closely related to concepts of plausibility and belief. The name “confidence interval” — rather than, for instance, the more accurate “coverage procedure” — encourages the Fundamental Confidence Fallacy.

The key confusion underlying the FCF is the confusion of what is known *before* observing the data — that the CI, whatever it will be, has a fixed chance of containing the true value — with what is known *after* observing the data. Frequentist CI theory says nothing at all about the probability that a particular, observed confidence interval contains the true value; it is either 0 (if the interval does not contain the parameter) or 1 (if the interval does contain the true value).

We offer several examples in this paper to show that what is known before computing an interval and what is known after computing it can be different. For now, we give a simple example, which we call the “trivial interval.” Consider the problem of estimating the mean of a continuous population with two independent observations, *y*_1_ and *y*_2_. If *y*_1_ > *y*_2_, we construct an confidence interval that contains all real numbers (−*∞*, *∞*); otherwise, we construct an empty confidence interval. The first interval is guaranteed to include the true value; the second is guaranteed not to. It is obvious that before observing the data, there is a 50 % probability that any sampled interval will contain the true mean. After observing the data, however, we know definitively whether the interval contains the true value. Applying the pre-data probability of 50 % to the post-data situation, where we know for certain whether the interval contains the true value, would represent a basic reasoning failure.

Post-data assessments of probability have never been an advertised feature of CI theory. Neyman, for instance, said “Consider now the case when a sample...is already drawn and the [confidence interval] given...Can we say that in this particular case the probability of the true value of [the parameter] falling between [the limits] is equal to [ *X*%]? The answer is obviously in the negative” (Neyman 1937, p. 349). According to frequentist philosopher Mayo (1981) “[the misunderstanding] seems rooted in a (not uncommon) desire for [...] confidence intervals to provide something which they cannot legitimately provide; namely, a measure of the degree of probability, belief, or support that an unknown parameter value lies in a specific interval.” Recent work has shown that this misunderstanding is pervasive among researchers, who likely learned it from textbooks, instructors, and confidence interval proponents (Hoekstra et al. 2014).

If confidence intervals cannot be used to assess the certainty with which a parameter is in a particular range, what can they be used for? Proponents of confidence intervals often claim that confidence intervals are useful for assessing the precision with which a parameter can be estimated. This is cited as one of the primary reasons confidence procedures should be used over null hypothesis significance tests (e.g., Cumming and Finch, 2005; Cumming, 2014; Fidler and Loftus, 2009; Loftus, 1993, 1996). For instance, Cumming (2014) writes that “[l]ong confidence intervals (CIs) will soon let us know if our experiment is weak and can give only imprecise estimates” (p. 10). Young and Lewis (1997) state that “[i]t is important to know how precisely the point estimate represents the true difference between the groups. The width of the CI gives us information on the precision of the point estimate” (p. 309). This is the second fallacy of confidence intervals, the “precision fallacy”:

### **Fallacy 2** (The Precision fallacy)

*The width of a confidence interval indicates the precision of our knowledge about the parameter. Narrow confidence intervals correspond to precise knowledge, while wide confidence errors correspond to imprecise knowledge.*

There is no necessary connection between the precision of an estimate and the size of a confidence interval. One way to see this is to imagine that two researchers — a senior researcher and a PhD student — are analyzing the data of 50 participants from an experiment. As an exercise for the PhD student’s benefit, the senior researcher decides to randomly divide the participants into two sets of 25 so that they can separately analyze half the data set. In a subsequent meeting, the two share with one another their Student’s *t* confidence intervals for the mean. The PhD student’s 95 % CI is 52±2, and the senior researcher’s 95 % CI is 53±4. The senior researcher notes that their results are broadly consistent, and that they could use the equally-weighted mean of their two respective point estimates, 52.5, as an overall estimate of the true mean.

The PhD student, however, argues that their two means should not be evenly weighted: she notes that her CI is half as wide and argues that her estimate is more precise and should thus be weighted more heavily. Her advisor notes that this cannot be correct, because the estimate from unevenly weighting the two means would be different from the estimate from analyzing the complete data set, which must be 52.5. The PhD student’s mistake is assuming that CIs directly indicate post-data precision. Later, we will provide several examples where the width of a CI and the uncertainty with which a parameter is estimated are in one case inversely related, and in another not related at all.

We cannot interpret observed confidence intervals as containing the true value with some probability; we also cannot interpret confidence intervals as indicating the precision of our estimate. There is a third common interpretation of confidence intervals: Loftus (1996), for instance, says that the CI gives an “indication of how seriously the observed pattern of means should be taken as a reflection of the underlying pattern of population means.” This logic is used when confidence intervals are used to test theory ( Velicer et al. 2008) or to argue for the null (or practically null) hypothesis (Loftus 1996). This is another fallacy of confidence interval that we call the “likelihood fallacy”.

### **Fallacy 3** (The Likelihood fallacy)

*A confidence interval contains the likely values for the parameter. Values inside the confidence interval are more likely than those outside. This fallacy exists in several varieties, sometimes involving plausibility, credibility, or reasonableness of beliefs about the parameter.*

A confidence procedure may have a fixed *average* probability of including the true value, but whether on any given sample it includes the “reasonable” values is a different question. As we will show, confidence intervals — even “good” confidence intervals, from a CI-theory perspective — can exclude almost all reasonable values, and can be empty or infinitesimally narrow, excluding all possible values (Blaker and Spjøtvoll 2000; Dufour 1997; Steiger 2004; Steiger and Fouladi 1997; Stock and Wright 2000). But Neyman (1941) writes,

“it is not suggested that we can ‘conclude’ that [the interval contains *θ*], nor that we should ‘believe’ that [the interval contains *θ*]...[we] *decide* to behave as if we actually knew that the true value [is in the interval]. This is done as a result of our decision and has nothing to do with ‘reasoning’ or ‘conclusion’. The reasoning ended when the [confidence procedure was derived]. The above process [of using CIs] is also devoid of any ‘belief’ concerning the value [...] of [*θ*].” (Neyman 1941, pp. 133–134)

It may seem strange to the modern user of CIs, but Neyman is quite clear that CIs do not support any sort of reasonable belief about the parameter. Even from a frequentist testing perspective where one accepts and rejects specific parameter values, Mayo and Spanos (2006) note that just because a specific value is in an interval does not mean it is warranted to accept it; they call this the “fallacy of acceptance.” This fallacy is analogous to accepting the null hypothesis in a classical significance test merely because it has not been rejected.

If confidence procedures do not allow an assessment of the probability that an interval contains the true value, if they do not yield measures of precision, and if they do not yield assessments of the likelihood or plausibility of parameter values, then what are they?

## The theory of confidence intervals

In a classic paper, Neyman (1937) laid the formal foundation for confidence intervals. It is easy to describe the practical problem that Neyman saw CIs as solving. Suppose a researcher is interested in estimating a parameter *θ*. Neyman suggests that researchers perform the following three steps:

- Perform an experiment, collecting the relevant data.
- Compute two numbers – the smaller of which we can call *L*, the greater *U* – forming an interval (*L*, *U*) according to a specified procedure.
- State that *L*<*θ*<*U* – that is, that *θ* is in the interval.

This recommendation is justified by choosing an procedure for step (b) such that in the long run, the researcher’s claim in step (c) will be correct, on average, *X*% of the time. A confidence interval is any interval computed using such a procedure.

We first focus on the meaning of the statement that *θ* is in the interval, in step (c). As we have seen, according to CI theory, what happens in step (c) is not a belief, a conclusion, or any sort of reasoning from the data. Furthermore, it is not associated with any level of uncertainty about whether *θ* is, actually, in the interval. It is merely a dichotomous statement that is meant to have a specified probability of being true in the long run.

Frequentist evaluation of confidence procedures is based on what can be called the “power” of the procedures, which is the frequency with which false values of a parameter are excluded. Better intervals are shorter on average, excluding false values more often (Lehmann 1959; Neyman 1937; 1941; Welch 1939). Consider a particular false value of the parameter, *θ*^′^ ≠ *θ*. Different confidence procedures will exclude that false value at different rates. If some confidence procedure CP *A* excludes *θ*^′^, on average, more often than some CP *B*, then CP *A* is better than CP *B* for that value.

Sometimes we find that one CP excludes *every* false value at a rate greater than some other CP; in this case, the first CP is uniformly more powerful than the second. There may even be a “best” CP: one that excludes every false *θ*^′^ value at a rate greater than any other possible CP. This is analogous to a most-powerful test. Although a best confidence procedure does not always exist, we can always compare one procedure to another to decide whether one is better in this way (Neyman, 1952). Confidence procedures are therefore closely related to hypothesis tests: confidence procedures control the rate of including the true value, and better confidence procedures have more power to exclude false values.

### Early skepticism

Skepticism about the usefulness of confidence intervals arose as soon as Neyman first articulated the theory (Neyman 1934).^2^ In the discussion of Neyman (1934), Bowley, pointing out what we call the fundamental confidence fallacy, expressed skepticism that the confidence interval answers the right question:

“I am not at all sure that the ‘confidence’ is not a ‘confidence trick.’ Does it really lead us towards what we need – the chance that in the universe which we are sampling the [parameter] is within these certain limits? I think it does not. I think we are in the position of knowing that either an improbable event has occurred or the [parameter] in the population is within the limits. To balance these things we must make an estimate and form a judgment as to the likelihood of the [parameter] in the universe [that is, a prior probability] – the very thing that is supposed to be eliminated.” (discussion of Neyman, 1934, p. 609)

In the same discussion, Fisher critiqued the theory for possibly leading to mutually contradictory inferences: “The [theory of confidence intervals] was a wide and very handsome one, but it had been erected at considerable expense, and it was perhaps as well to count the cost. The first item to which he [Fisher] would call attention was the loss of uniqueness in the result, and the consequent danger of apparently contradictory inferences.” (discussion of Neyman, 1934, p. 618; see also Fisher, 1935). Though, as we will see, the critiques are accurate, in a broader sense they missed the mark. Like modern proponents of confidence intervals, the critics failed to understand that Neyman’s goal was different from theirs: Neyman had developed a behavioral theory designed to control error rates, not a theory for reasoning from data (Neyman 1941).

In spite of the critiques, confidence intervals have grown in popularity to be the most widely used interval estimators. Alternatives — such as Bayesian credible intervals and Fisher’s fiducial intervals — are not as commonly used. We suggest that this is largely because people do not understand the differences between confidence interval, Bayesian, and fiducial theories, and how the resulting intervals cannot be interpreted in the same way. In the next section, we will demonstrate the logic of confidence interval theory by building several confidence procedures and comparing them to one another. We will also show how the three fallacies affect inferences with these intervals.

## Example 1: The lost submarine

In this section, we present an example taken from the confidence interval literature (Berger and Wolpert 1988; Lehmann 1959; Pratt 1961; Welch 1939) designed to bring into focus how CI theory works. This example is intentionally simple; unlike many demonstrations of CIs, no simulations are needed, and almost all results can be derived by readers with some training in probability and geometry. We have also created interactive versions of our figures to aid readers in understanding the example; see the figure captions for details.

A 10-meter-long research submersible with several people on board has lost contact with its surface support vessel. The submersible has a rescue hatch exactly halfway along its length, to which the support vessel will drop a rescue line. Because the rescuers only get one rescue attempt, it is crucial that when the line is dropped to the craft in the deep water that the line be as close as possible to this hatch. The researchers on the support vessel do not know where the submersible is, but they do know that it forms two distinctive bubbles. These bubbles could form anywhere along the craft’s length, independently, with equal probability, and float to the surface where they can be seen by the support vessel.

**Fig. 1:**
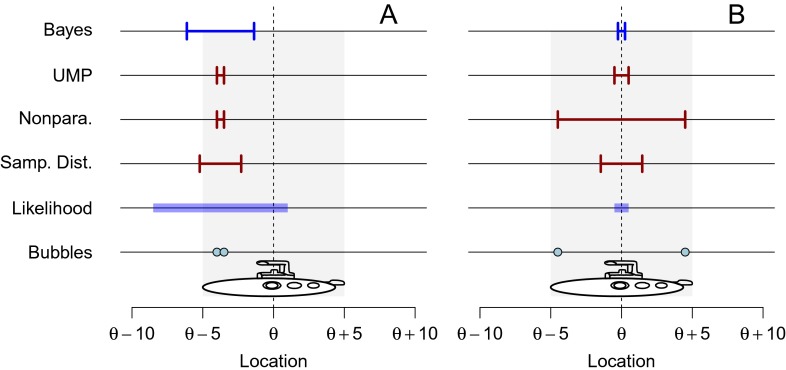
Submersible rescue attempts. Note that likelihood and CIs are depicted from bottom to top in the order in which they are described in the text. See text for details. An interactive version of this figure is available at http://learnbayes.org/redirects/CIshiny1.html

The situation is shown in Fig. 1a. The rescue hatch is the unknown location *θ*, and the bubbles can rise from anywhere with uniform probability between *θ*−5 meters (the bow of the submersible) to *θ*+5 meters (the stern of the submersible). The rescuers want to use these bubbles to infer where the hatch is located. We will denote the first and second bubble observed by *y*_1_ and *y*_2_, respectively; for convenience, we will often use *x*_1_ and *x*_2_ to denote the bubbles ordered by location, with *x*_1_ always denoting the smaller location of the two. Note that $\bar {y}=\bar {x}$, because order is irrelevant when computing means, and that the distance between the two bubbles is |*y*_1_−*y*_2_|=*x*_2_−*x*_1_. We denote this difference as *d*.

The rescuers first note that from observing two bubbles, it is easy to rule out all values except those within five meters of both bubbles because no bubble can occur further than 5 meters from the hatch. If the two bubble locations were *y*_1_ = 4 and *y*_2_ = 6, then the possible locations of the hatch are between 1 and 9, because only these locations are within 5 meters of both bubbles. This constraint is formally captured in the *likelihood*, which is the joint probability density of the observed data for all possible values of *θ*. In this case, because the observations are independent, the joint probability density is:

$$\begin{array}{@{}rcl@{}} p(y_{1}, y_{2}; \theta) &=& p_{y}(y_{1};\theta)\times p_{y}(y_{2};\theta). \end{array} $$

The density for each bubble *p*_*y*_ is uniform across the submersible’s 10 meter length, which means the joint density must be 1/10×1/10=1/100. If the lesser of *y*_1_ and *y*_2_ (which we denote *x*_1_) is greater than *θ*−5, then obviously both *y*_1_ and *y*_2_ must be greater than *θ*−5. This means that the density, written in terms of constraints on *x*_1_ and *x*_2_, is:

1 $$\begin{array}{@{}rcl@{}} p(y_{1}, y_{2}; \theta) &=& \left\{\begin{array}{ll} 1/100, &\text{if}~x_{1}>\theta-5~\text{and}~x_{2}<\theta+5,\\ 0& \text{otherwise}. \end{array} \right. \end{array} $$

If we write Eq 1 as a function of the unknown parameter *θ* for fixed, observed data, we get the likelihood, which indexes the information provided by the data about the parameter. In this case, it is positive only when a value *θ* is possible given the observed bubbles (see also Figs. 1 and 5):

$$\begin{array}{@{}rcl@{}} p(\theta ; y_{1}, y_{2}) &=& \left\{\begin{array}{cl} 1,&\theta>x_{2} - 5~\text{and}~\theta\leq x_{1}+5,\\ 0& \text{otherwise}. \end{array}\right.\\ \end{array} $$

We replaced 1/100 with 1 because the particular values of the likelihood do not matter, only their relative values. Writing the likelihood in terms of $\bar {x}$ and the difference between the bubbles *d* = *x*_2_−*x*_1_, we get an interval:

2 $$\begin{array}{@{}rcl@{}} p(\theta ; y_{1}, y_{2})\! &=&\!\!\left\{\!\begin{array}{ll} 1,&\! \bar{x} - (5 - d/2) < \theta \leq \bar{x} + (5 - d/2),\\ 0& \!\text{otherwise}. \end{array}\right. \end{array} $$

If the likelihood is positive, the value *θ* is possible; if it is 0, that value of *θ* is impossible. Expressing the likelihood as in Eq. 2 allows us to see several important things. First, the likelihood is centered around a reasonable point estimate for *θ*, $\bar {x}$. Second, the width of the likelihood 10−*d*, which here is an index of the uncertainty of the estimate, is larger when the difference between the bubbles *d* is smaller. When the bubbles are close together, we have little information about *θ* compared to when the bubbles are far apart. Keeping in mind the likelihood as the information in the data, we can define our confidence procedures.

### Five confidence procedures

A group of four statisticians^3^ happen to be on board, and the rescuers decide to ask them for help improving their judgments using statistics. The four statisticians suggest four different 50 % confidence procedures. We will outline the four confidence procedures; first, we describe a trivial procedure that no one would ever suggest. An applet allowing readers to sample from these confidence procedures is available at the link in the caption for Fig. 1.

#### 0. A trivial procedure

A trivial 50 % confidence procedure can be constructed by using the ordering of the bubbles. If *y*_1_ > *y*_2_, we construct an interval that contains the whole ocean, (−*∞*, *∞*). If *y*_2_ > *y*_1_, we construct an interval that contains only the single, *exact* point directly under the middle of the rescue boat. This procedure is obviously a 50 % confidence procedure; exactly half of the time — when *y*_1_ > *y*_2_ — the rescue hatch will be within the interval. We describe this interval merely to show that *by itself*, a procedure including the true value *X* % of the time means nothing (see also Basu, 1981). We must obviously consider something more than the confidence property, which we discuss subsequently.

#### 1. A procedure based on the sampling distribution of the mean

The first statistician suggests building a confidence procedure using the sampling distribution of the mean $\bar {x}$. The sampling distribution of $\bar {x}$ has a known triangular distribution with *θ* as the mean. With this sampling distribution, there is a 50 % probability that $\bar {x}$ will differ from *θ* by less than $5 - 5/\sqrt {2}$, or about 1.46m. We can thus use $\bar {x} - \theta $ as a so-called “pivotal quantity” (Casella & Berger, 2002; see the supplement to this manuscript for more details) by noting that there is a 50 % probability that *θ* is within this same distance of $\bar {x}$ in repeated samples. This leads to the confidence procedure

$$\bar{x} \pm \left( 5 - 5/\sqrt{2}\right), $$

which we call the “sampling distribution” procedure. This procedure also has the familiar form $\bar {x} \pm C\times SE$, where here the standard error (that is, the standard deviation of the estimate $\bar {x}$) is known to be 2.04.

#### 2. A nonparametric procedure

The second statistician notes that *θ* is both the mean and median bubble location. Olive (2008) and Rusu and Dobra (2008) suggest a nonparametric confidence procedure for the median that in this case is simply the interval between the two observations:

$$\bar{x} \pm d/2. $$

It is easy to see that this must be a 50 % confidence procedure; the probability that both observations fall below *θ* is .5×.5=.25, and likewise for both falling above. There is thus a 50 % chance that the two observations encompass *θ*. Coincidentally, this is the same as the 50 % Student’s *t* procedure for *n* = 2.

#### 3. The uniformly most-powerful procedure

The third statistician, citing Welch (1939), describes a procedure that can be thought of as a slight modification of the nonparametric procedure. Suppose we obtain a particular confidence interval using the nonparametric procedure. If the nonparametric interval is more than 5 meters wide, then it *must* contain the hatch, because the only possible values are less than 5 meters from both bubbles. Moreover, in this case the interval will contain impossible values, because it will be wider than the likelihood. We can exclude these impossible values by truncating the interval to the likelihood whenever the width of the interval is greater than 5 meters:

$$\bar{x} \pm \left\{\begin{array}{lllr} \frac{d}{2} & \text{if} & d < 5 & \text{(nonparametric procedure)}\\ 5 - \frac{d}{2} &\text{if} & d \geq 5 & \text{(likelihood)} \end{array} \right. $$

This will not change the probability that the interval contains the hatch, because it is simply replacing one interval that is sure to contain it with another. Pratt (1961) noted that this interval can be justified as an inversion of the uniformly most-powerful (UMP) test.

#### 4. An objective Bayesian procedure

The fourth statistician suggests an objective Bayesian procedure. Using this procedure, we simply take the central 50 % of the likelihood as our interval:

$$\bar{x} \pm \frac{1}{2}\left( 5 - \frac{d}{2}\right). $$

From the objective Bayesian viewpoint, this can be justified by assuming a prior distribution that assigns equal probability to each possible hatch location. In Bayesian terms, this procedure generates “credible intervals” for this prior. It can also be justified under Fisher’s fiducial theory; see Welch (1939).

### Properties of the procedures

The four statisticians report their four confidence procedures to the rescue team, who are understandably bewildered by the fact that there appear to be at least four ways to infer the hatch location from two bubbles. Just after the statisticians present their confidence procedures to the rescuers, two bubbles appear at locations *x*_1_ = 1 and *x*_2_ = 1.5. The resulting likelihood and the four confidence intervals are shown in Fig. 1a.

#### The fundamental confidence fallacy

After using the observed bubbles to compute the four confidence intervals, the rescuers wonder how to interpret them. It is clear, first of all, why the fundamental confidence fallacy is a fallacy. As Fisher pointed out in the discussion of CI theory mentioned above, for any given problem — as for this one — there are many possible confidence procedures. These confidence procedures will lead to different confidence intervals. In the case of our submersible confidence procedures, all confidence intervals are centered around $\bar {x}$, and so the intervals will be nested within one another.

If we mistakenly interpret these observed intervals as having a 50 % probability of containing the true value, a logical problem arises. First, there must always be a 50 % probability that the *shortest* interval contains the parameter. The reason is basic probability theory: the narrowest interval would have probability 50 % of including the true value, and the widest interval would have probability 50 % of excluding the true value. According to this reasoning, there must be a 0 % probability that the true value is outside the narrower, nested interval yet inside the wider interval. If we believed the FCF, we would always come to the conclusion that the shortest of a set of nested *X*% intervals has an *X*% probability of containing the true value. Of course, the confidence procedure “always choose the shortest of the nested intervals” will tend to have a lower than *X*% probability of including the true value. If we believed the FCF, then we must come to the conclusion that the shortest interval simultaneously has an *X*% probability of containing the true value, and a less than *X*% probability. Believing the FCF results in contradiction.

This point regarding the problem of interpreting nested CIs is not, by itself, a critique of confidence interval theory *proper*; it is rather a critique of the folk theory of confidence. Neyman himself was very clear that this interpretation was not permissible, using similarly nested confidence intervals to demonstrate the fallacy (Neyman 1941, pp. 213–215). It is a warning that the improper interpretations of confidence intervals used throughout the scientific literature leads to mutually contradictory inferences, just as Fisher warned.

Even without nested confidence procedures, one can see that the FCF must be a fallacy. Consider Fig. 1b, which shows the resulting likelihood and confidence intervals when *x*_1_ = 0.5 and *x*_2_ = 9.5. When the bubbles are far apart, as in this case, the hatch can be localized very precisely: the bubbles are far enough apart that they must have come from the bow and stern of the submersible. The sampling distribution, nonparametric, and UMP confidence intervals all encompass the likelihood, meaning that there is 100 % certainty that these 50 % confidence intervals contain the hatch. Reporting 50 % certainty, 50 % probability, or 50 % confidence in a specific interval that surely contains the parameter would clearly be a mistake.

#### Relevant subsets

**Fig. 2:**
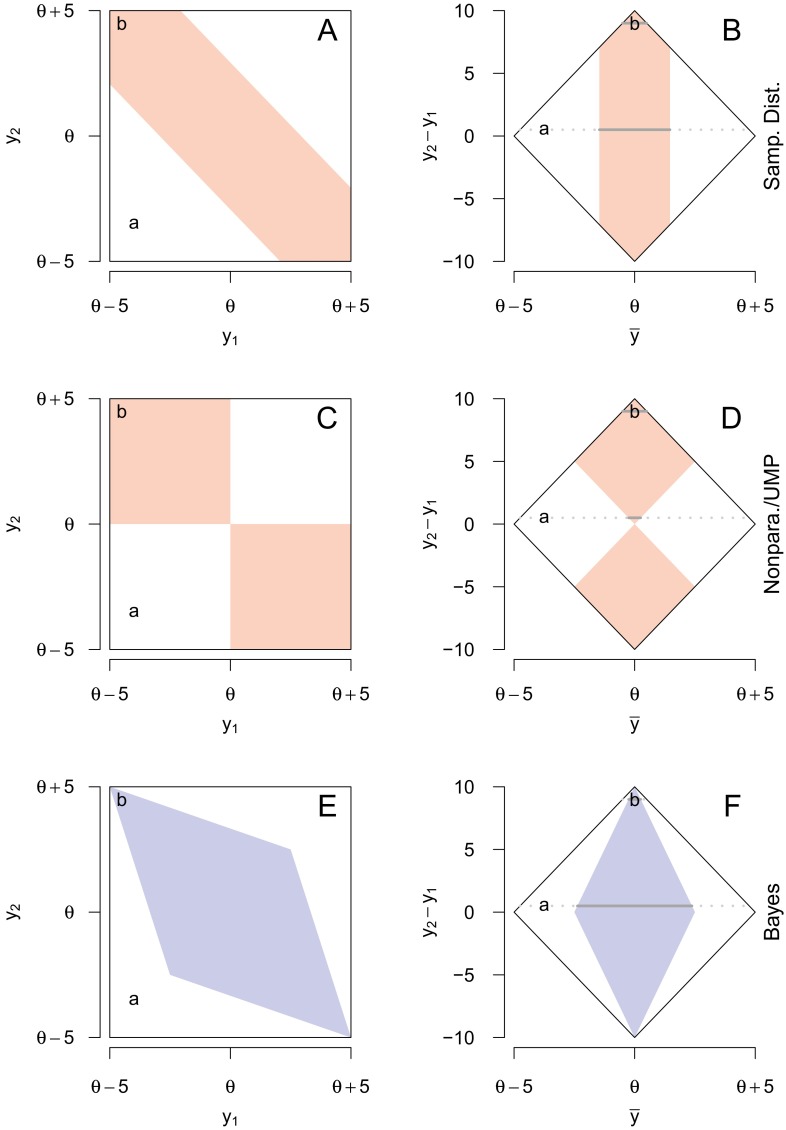
*Left*: Possible locations of the first (*y*_1_) and second (*y*_2_) bubbles. *Right*: *y*_2_−*y*_1_ plotted against the mean of *y*_1_ and *y*_2_. *Shaded regions* show the areas where the respective 50 % confidence interval contains the true value. The figures in the *top row* (**a, b**) show the sampling distribution interval; the *middle row* (**c, d**) shows the NP and UMP intervals; the *bottom row* (**e, f**) shows the Bayes interval. Points ‘a’ and ‘b’ represent the pairs of bubbles from Fig. 1a and b, respectively. An interactive version of this figure is available at http://learnbayes.org/redirects/CIshiny1.html

The fact that we can have 100 % certainty that a 50 % CI contains the true value is a specific case of a more general problem flowing from the FCF. The shaded regions in Fig. 2, left column, show when the true value is contained in the various confidence procedures for all possible pairs of observations. The top, middle, and bottom row correspond to the sampling distribution, nonparametric/UMP, and the Bayes procedures, respectively. Because each procedure is a 50 % confidence procedure, in each plot the shaded area occupies 50 % of the larger square delimiting the possible observations. The points ‘a’ and ‘b’ are the bubble patterns in Fig. 1a and b, respectively; point ‘b’ is in the shaded region for each intervals because the true value is included in every kind of interval, as shown in Fig. 1b; likewise, ‘a’ is outside every shaded region because all CIs exclude the true value for this observed bubble pair.

Instead of considering the bubbles themselves, we might also translate their locations into the mean location $\bar {y}$ and the difference between them, *b* = *y*_2_−*y*_1_. We can do this without loss of any information: $\bar {y}$ contains the point estimate of the hatch location, and *b* contains the information about the precision of that estimate. Figure 2, right column, shows the same information as in the left column, except as a function of $\bar {y}$ and *b*. The figures in the right column are 45^∘^ clockwise rotations of those in the left. Although the two columns show the same information, the rotated right column reveals a critical fact: the various confidence procedures have different probabilities of containing the true value when the distance between the bubbles varies.

To see this, examine the horizontal line under point ‘a’ in Fig. 2b. The horizontal line is the subset of all bubble pairs that show the same difference between the bubbles as those in Fig. 1a: 0.5 meters. About 31 % of this line falls under the shaded region, meaning that in the long run, 31 % of sampling distributions intervals will contain the true value, when the bubbles are 0.5 meters apart. For the nonparametric and UMP intervals (middle row), this percentage is only about 5 %. For the Bayes interval (bottom row), it is exactly 50 %.

Believing the FCF implies believing that we can use the long-run probability that a procedure contains the true value as an index of our post-data certainty that a particular interval contains the true value. But in this case, we have identified *two* long-run probabilities for each interval: the average long-run probability *not* taking into account the observed difference — that is, 50 % — and the long-run probability taking into account *b* which, for the sampling distribution interval is 31 % and for the nonparametric/UMP intervals is 5 %. Both are valid long-run probabilities; which do we use for our inference? Under FCF, both are valid. Hence the FCF leads to contradiction.

The existence of multiple, contradictory long-run probabilities brings back into focus the confusion between what we know before the experiment with what we know after the experiment. For any of these confidence procedures, we know before the experiment that 50 % of future CIs will contain the true value. After observing the results, conditioning on a known property of the data — such as, in this case, the variance of the bubbles — can radically alter our assessment of the probability.

The problem of contradictory inferences arising from multiple applicable long-run probabilities is an example of the “reference class” problem (Venn, 1888; Reichenbach, 1949), where a single observed event (e.g., a CI) can be seen as part of several long-run sequences, each with a different long-run probability. Fisher noted that when there are identifiable subsets of the data that have different probabilities of containing the true value — such as those subsets with a particular value of *d*, in our confidence interval example — those subsets are relevant to the inference (Fisher 1959). The existence of relevant subsets means that one can assign more than one probability to an interval. Relevant subsets are identifiable in many confidence procedures, including the common classical Student’s *t* interval, where wider CIs have a greater probability of containing the true value (Buehler, 1959; Buehler & Feddersen,^1963^; Casella, 1992; Robinson, 1979). There are, as far as we know, only two general strategies for eliminating the threat of contradiction from relevant subsets: Neyman’s strategy of avoiding any assignment of probabilities to particular intervals, and the Bayesian strategy of always conditioning on the observed data, to be discussed subsequently.

#### The precision and likelihood fallacies

**Fig. 3:**
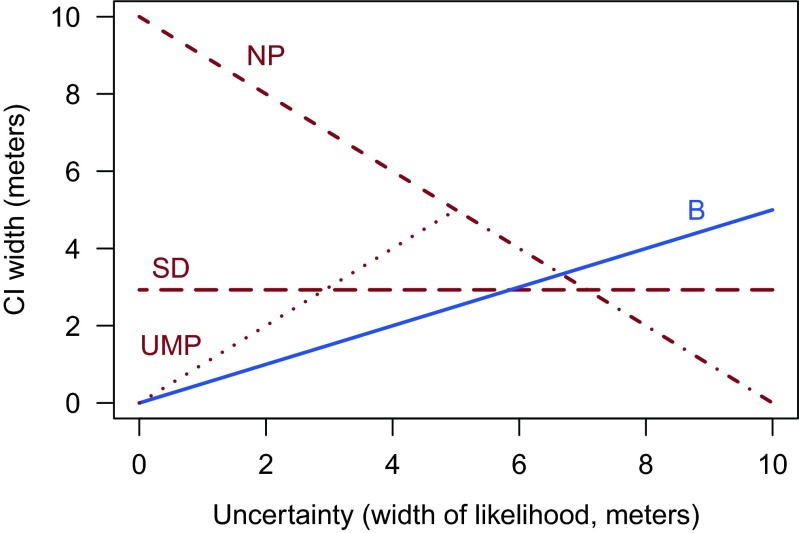
The relationship between CI width and the uncertainty in the estimation of the hatch location for the four confidence procedures. SD: Sampling distribution procedure; NP: Nonparametric procedure; UMP: UMP procedure; B: Bayes procedure. Note that the NP and UMP procedures overlap when the width of the likelihood is >5. An interactive version of this figure is available at http://learnbayes.org/redirects/CIshiny1.html

This set of confidence procedures also makes clear the precision fallacy. Consider Fig. 3, which shows how the width of each of the intervals produced by the four confidence procedures changes as a function of the width of the likelihood. The Bayes procedure tracks the uncertainty in the data: when the likelihood is wide, the Bayes CI is wide. The reason for this necessary correspondence between the likelihood and the Bayes interval will be discussed later.

Intervals from the sampling distribution procedure, in contrast, have a fixed width, and so cannot reveal any information about the precision of the estimate. The sampling distribution interval is of the commonly-seen CI form

$$\bar{x}\pm C\times SE, $$

Like the CI for a normal population mean with known population variance, the standard error — defined as the standard deviation of the sampling distribution of $\bar {x}$ — is known and fixed; here, it is approximately 2.04 (see the supplement for details). This indicates that the long-run standard error — and hence, confidence intervals based on the standard error — cannot always be used as a guide to the uncertainty we should have in a parameter estimate.

Strangely, the nonparametric procedure generates intervals whose widths are *inversely* related to the uncertainty in the parameter estimates. Even more strangely, intervals from the UMP procedure initially increase in width with the uncertainty in the data, but when the width of the likelihood is greater than 5 meters, the width of the UMP interval is inversely related to the uncertainty in the data, like the nonparametric interval. This can lead to bizarre situations. Consider observing the UMP 50 % interval [1,1.5]. This is consistent with two possible sets of observations: (1,1.5), and (−3.5,6). Both of these sets of bubbles will lead to the same CI. Yet the second data set indicates high precision, and the first very low precision! The UMP and sampling distribution procedures share the dubious distinction that their CIs cannot be used to work backwards to the observations. In spite of being the “most powerful” procedure, the UMP procedure clearly throws away important information.

To see how the likelihood fallacy is manifest in this example, consider again Fig. 3. When the uncertainty is high, the likelihood is wide; yet the nonparametric and UMP intervals are extremely narrow, indicating both false precision and excluding almost all likely values. Furthermore, the sampling distribution procedure and the nonparametric procedure can contain impossible values.^4^

### Evaluating the confidence procedures

The rescuers who have been offered the four intervals above have a choice to make: which confidence procedure to choose? We have shown that several of the confidence procedures have counter-intuitive properties, but thus far, we have not made any firm commitments about which confidence procedures should be preferred to the others. For the sake of our rescue team, who have a decision to make about which interval to use, we now compare the four procedures directly. We begin with the evaluation of the procedures from the perspective of confidence interval theory, then evaluate them according to Bayesian theory.

**Fig. 4:**
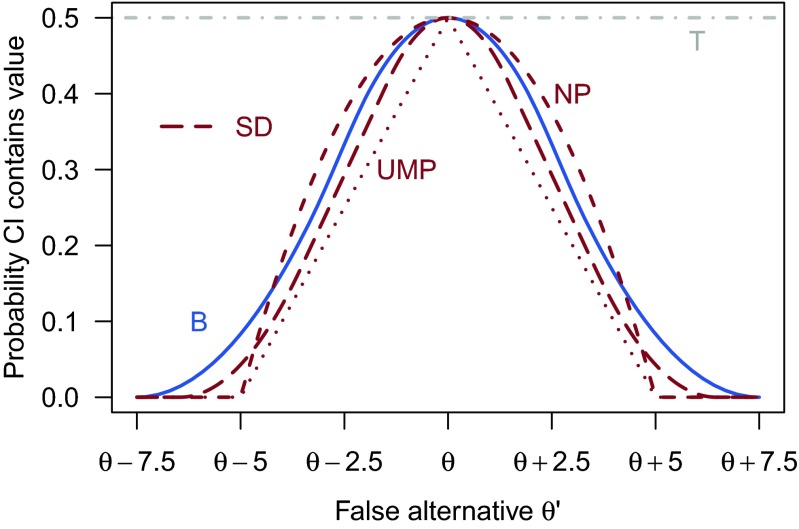
The probability that each confidence procedure includes false values $\theta ^{\prime }$. T: Trivial procedure; SD: Sampling distribution procedure NP: Nonparametric procedure; UMP: UMP procedure; B: Bayes procedure. The line for the sampling distribution procedure (*dashed line*) is between the lines for the Bayes procedure and the UMP procedure. An interactive version of this figure is available at http://learnbayes.org/redirects/CIshiny1.html

As previously mentioned, confidence interval theory specifies that better intervals will include false values less often. Figure 4 shows the probability that each of the procedures include a value *θ*^′^ at a specified distance from the hatch *θ*. All procedures are 50 % confidence procedures, and so they include the true value *θ* 50 % of the time. Importantly, however, the procedures include particular false values *θ*^′^≠*θ* at different rates. See the interactive versions of Figs. 1*and*4 linked in the figure captions for a hands-on demonstration.

The trivial procedure (T; gray horizontal line) is obviously a bad interval because it includes every false value with the same frequency as the true value. This is analogous to a hypothesis test with power equal to its Type I error rate. The trivial procedure will be worse than any other procedure, unless the procedure is specifically constructed to be pathological. The UMP procedure (UMP), on the other hand, is better than every other procedure for every value of *θ*^′^. This is due to the fact that it was created by inverting a most-powerful test.

The ordering among the three remaining procedures can be seen by comparing their curves. The sampling distribution procedure (SD) is always superior to the Bayes procedure (B), but not to the nonparametric procedure (NP). The nonparametric procedure and the Bayes procedure curves overlap, so one is not preferred to the other. Welch (1939) remarked that the Bayes procedure is “not the best way of constructing confidence limits” using precisely the frequentist comparison shown in Fig. 4 with the UMP interval.^5^

The frequentist comparison between the procedures is instructive, because we have arrived at an ordering of the procedures employing the criteria suggested by Neyman and used by the modern developers of new confidence procedures: coverage and power. The UMP procedure is the best, followed by the sampling distribution procedure. The sampling distribution procedure is better than the Bayes procedure. The nonparametric procedure is not preferred to any interval, but neither is it the worst.

We can also examine the procedures from a Bayesian perspective, which is primarily concerned with whether the inferences are reasonable in light of the data and what was known before the data were observed (Howson and Urbach 2006). We have already seen that interpreting the non-Bayesian procedures in this way leads to trouble, and that the Bayesian procedure, unsurprisingly, has better properties in this regard. We will show how the Bayesian interval was derived in order to provide insight into why it has good properties.

**Fig. 5:**
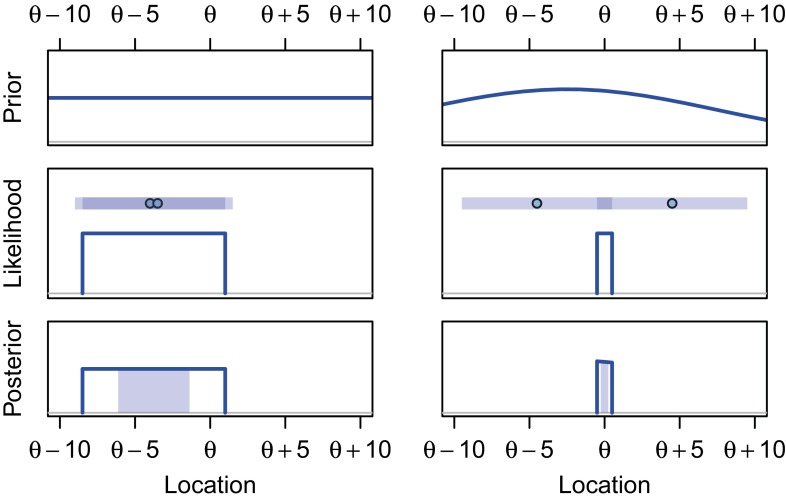
Forming Bayesian credible intervals. Prior information (*top*) is combined with the likelihood information from the data (*middle*) to yield a posterior distribution (*bottom*). In the likelihood plots, the shaded regions show the locations within 5 meters of each bubble; the *dark shaded* regions show where these overlap, indicating the possible location of the hatch *θ*. In the posterior plots, the central 50 % region (*shaded region* within posterior) shows one possible 50 % credible interval, the central credible interval. An interactive version of this figure is available at http://learnbayes.org/redirects/CIshiny1.html

Consider the left column of Fig. 5, which shows Bayesian reasoning from prior and likelihood to posterior and so-called credible interval. The prior distribution in the top panel shows that before observing the data, all the locations in this region are equally probable. Upon observing the bubbles shown in Fig. 1a — also shown in the top of the “likelihood” panel — the likelihood is a function that is 1 for all possible locations for the hatch, and 0 otherwise. To combine our prior knowledge with the new information from the two bubbles, we condition what we knew before on the information in the data by multiplying by the likelihood — or, equivalently, excluding values we know to be impossible — which results in the posterior distribution in the bottom row. The central 50 % credible interval contains all values in the central 50 % of the area of the posterior, shown as the shaded region. The right column of Fig. 5 shows a similar computation using an informative prior distribution that does not assume that all locations are equally likely, as might occur if some other information about the location of the submersible were available.

It is now obvious why the Bayesian credible interval has the properties typically ascribed to confidence intervals. The credible interval can be interpreted as having a 50 % probability of containing the true value, because the values within it account for 50 % of the posterior probability. It reveals the precision of our knowledge of the parameter, in light of the data and prior, through its relationship with the posterior and likelihood.

Of the five procedures considered, intervals from the Bayesian procedure are the *only ones* that can be said to have 50 % probability of containing the true value, upon observing the data. Importantly, the ability to interpret the interval in this way arises from Bayesian theory and not from confidence interval theory. Also importantly, it was necessary to stipulate a prior to obtain the desired interval; the interval should be interpreted in light of the stipulated prior. Of the other four intervals, none can be justified as providing a “reasonable” inference or conclusion from the data, because of their strange properties and that there is no possible prior distribution that could lead to these procedures. In this light, it is clear why Neyman’s rejection of “conclusions” and “reasoning” from data naturally flowed from his theory: the theory, after all, does not support such ideas. It is also clear that if they care about making reasonable inferences from data, scientists might want want to reject confidence interval theory as a basis for evaluating procedures.

We can now review what we know concerning the four procedures. Only the Bayesian procedure — when its intervals are interpreted as credible intervals — allows the interpretation that there is a 50 % probability that the hatch is located in the interval. Only the Bayesian procedure properly tracks the precision of the estimate. Only the Bayesian procedure covers the plausible values in the expected way: the other procedures produce intervals that are *known* with certainty — by simple logic — to contain the true value, but still are “50 %” intervals. The non-Bayesian intervals have undesirable, even bizarre properties, which would lead any reasonable analyst to reject them as a means to draw inferences. Yet the Bayesian procedure is judged by frequentist CI theory as inferior.

The disconnect between frequentist theory and Bayesian theory arises from the different goals of the two theories. Frequentist theory is a “pre-data” theory. It looks forward, devising procedures that will have particular average properties in repeated sampling (Jaynes 2003; Mayo 1981; 1982) in the future (see also Neyman, 1937, p. 349). This thinking can be clearly seen in Neyman (1941) as quoted above: reasoning ends once the procedure is derived. Confidence interval theory is vested in the average frequency of including or excluding true and false parameter values, respectively. Any given inference may — or may not — be reasonable in light of the observed data, but this is not Neyman’s concern; he disclaims any conclusions or beliefs on the basis of data. Bayesian theory, on the other hand, is a post-data theory: a Bayesian analyst uses the information in the data to determine what is reasonable to believe, in light of the model assumptions and prior information.

Using an interval justified by a pre-data theory to make post-data inferences can lead to unjustified, and possibly arbitrary, inferences. This problem is not limited to the pedagogical submersible example (Berger and Wolpert 1988; Wagenmakers et al. 2014) though this simple example is instructive for identifying these issues. In the next section we show how a commonly-used confidence interval leads to similarly flawed post-data inferences.

## Example 2: A confidence interval in the wild

The previous example was designed to show, in an accessible example, the logic of confidence interval theory. Further, it showed that confidence procedures cannot be assumed to have the properties that analysts desire.

When presenting the confidence intervals, CI proponents almost always focus on estimation of the mean of a normal distribution. In this simple case, frequentist and Bayesian (with a “non-informative” prior) answers numerically coincide.^6^ However, the proponents of confidence intervals suggest the use of confidence intervals for many other quantities: for instance, standardized effect size Cohen’s *d* (Cumming and Finch 2001), medians (Bonett and Price 2002; Olive 2008), correlations (Zou 2007), ordinal association (Woods 2007), and many others. Quite often authors of such articles provide no analysis of the properties of the proposed confidence procedures beyond showing that they contain the true value in the correct proportion of samples: that is, that they are confidence procedures. Sometimes the authors provide an analysis of the frequentist properties of the procedures, such as average width. The developers of new confidence procedures do not, as a rule, examine whether their procedures allow for valid post-data reasoning.

As the first example showed, a sole focus on frequentist properties of procedures is potentially disastrous for users of these confidence procedures because a confidence procedure has no guarantee of supporting reasonable inferences about the parameter of interest. Casella (1992) underscores this point with confidence intervals, saying that “we must remember that practitioners are going to make conditional (post-data) inferences. Thus, we must be able to assure the user that any inference made, either pre-data or post-data, possesses some definite measure of validity” (p. 10). Any development of an interval procedure that does not, at least in part, focus on its post-data properties is incomplete at best and extremely misleading at worst: *caveat emptor*.

Can such misleading inferences occur using procedures suggested by proponents of confidence intervals, and in use by researchers? The answer is yes, which we will show by examining a confidence interval for *ω*^2^, the proportion of variance accounted for in ANOVA designs. The parameter *ω*^2^ serves as a measure of effect size when there are more than two levels in a one-way design. This interval was suggested by Steiger (2004, see also Steiger & Fouladi,^1997^), cited approvingly by Cumming (2014), implemented in software for social scientists (e.g., Kelley, 2007a, b), and evaluated, solely for its frequentist properties, by Finch and French (2012). The problems we discuss here are shared by other related confidence intervals, such as confidence intervals for *η*^2^, partial *η*^2^, the noncentrality parameter of the *F* distribution, the signal-to-noise ratio *f*, RMSSE Ψ, and others discussed by Steiger (2004).

Steiger (2004) introduces confidence intervals by emphasizing a desire to avoid significance tests, and to focus more on the precision of estimates. Steiger says that “the scientist is more interested in knowing how large the difference between the two groups is (and how precisely it has been determined) than whether the difference between the groups is 0” (pp. 164-165). Steiger and Fouladi (1997) say that “[t]he advantage of a confidence interval is that the width of the interval provides a ready indication of the precision of measurement...” (p. 231). Given our knowledge of the precision fallacy these statements should raise a red flag.

Steiger then offers a confidence procedure for *ω*^2^ by inverting a significance test. Given the strange behavior of the UMP procedure in the submersible example, this too should raise a red flag. A confidence procedure based on a test — even a good, high-powered test — will not in general yield a procedure that provides for reasonable inferences. We will outline the logic of building a confidence interval by inverting a significance test before showing how Steiger’s confidence interval behaves with data.

To understand how a confidence interval can be built by inverting a significance test, consider that a two-sided significance test of size *α* can be thought of as a combination of two one-sided tests at size *α*/2: one for each tail. The two-sided test rejects when one of the one-tailed tests rejects. To build a 68 % confidence interval (i.e., an interval that covers the true value as often as the commonly-used standard error for the normal mean), we can use two one-sided tests of size (1−.68)/2=.16. Suppose we have a one-way design with three groups and *N* = 10 participants in each group. The effect size *ω*^2^ in such a design indexes how large *F* will be: larger *ω*^2^ values tend to yield larger *F* values. The distribution of *F* given the effect size *ω*^2^ is called the noncentral *F* distribution. When *ω*^2^ = 0 — that is, there is no effect — the familiar central *F* distribution is obtained.

**Fig. 6:**
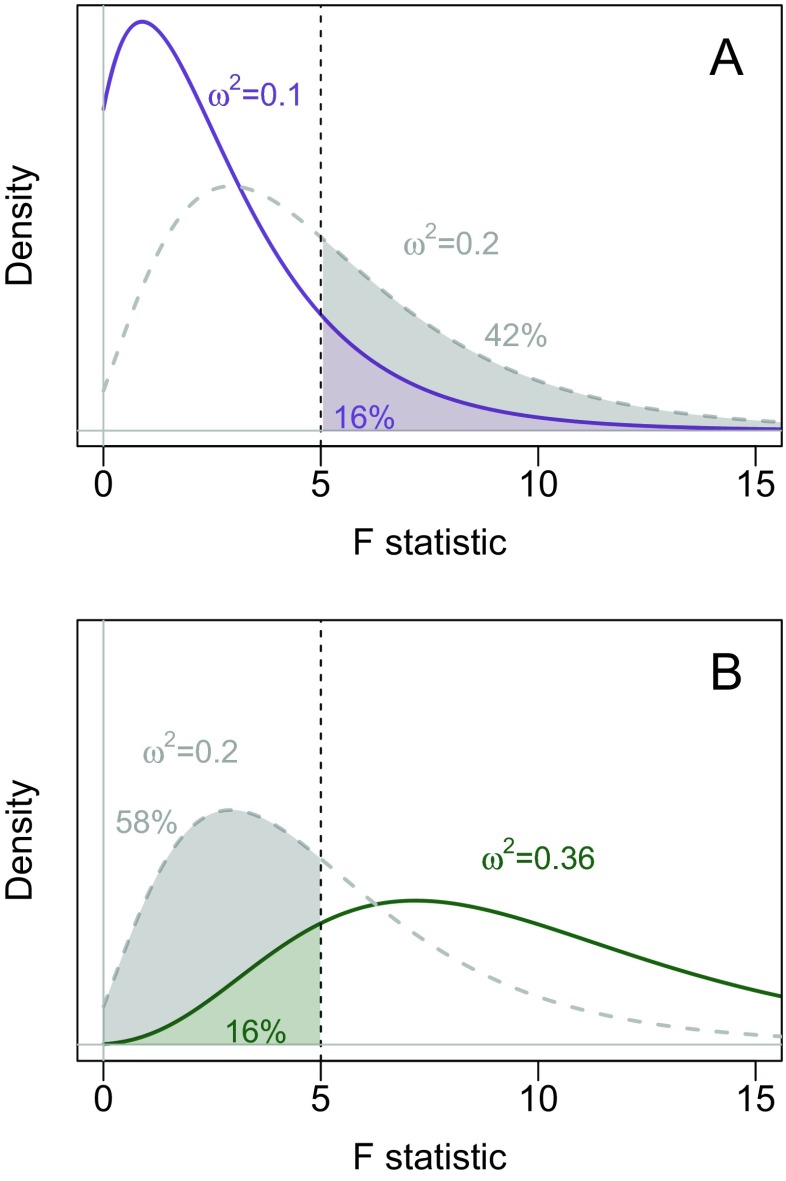
Building a confidence interval by inverting a significance test. A: Two noncentral *F* distributions, with true *ω* ^2^ = .1 (*blue solid line*) and true *ω* ^2^ = .2 (*dashed gray line*). When *F*(2,27)=5, the upper-tailed *p* value for these tests are .16 and .42, respectively. B: Two noncentral *F* distributions, with true *ω* ^2^ = .36 (*red solid line*) and true *ω* ^2^ = .2 (*dashed gray line*). When *F*(2,27)=5, the lower-tailed *p* value for these tests are .16 and .58, respectively

Consider first a one-sided test that rejects when *F* is large. Figure 6a shows that a test of the null hypothesis that *ω*^2^ = .1 would yield *p* = .16 when *F*(2,27)=5. If we tested larger values of *ω*^2^, the *F* value would not lead to a rejection; if we tested smaller values of *ω*^2^, they would be rejected because their *p* values would be below .16. The gray dashed line in Fig. 6a shows the noncentral *F*(2,27) distribution for *ω*^2^ = .2; it is apparent that the *p* value for this test would be greater than .16, and hence *ω*^2^ = .2 would not be rejected by the upper-tailed test of size .16. Now consider the one-sided test that rejects when *F* is small. Figure 6b shows that a test of the null hypothesis that *ω*^2^ = .36 would yield *p* = .16 when *F*(2,27)=5; any *ω*^2^ value greater than .36 would be rejected with *p*<.16, and any *ω*^2^ value less than .36 would not.

Considering the two one-tailed tests together, for any *ω*^2^ value in [.1,.36], the *p* value for both one-sided tests will be greater than *p* > .16 and hence will not lead to a rejection. A 68 % confidence interval for when *F*(2,27)=5 can be defined as all *ω*^2^ values that are not rejected by either of the two-tailed tests, and so [.1,.36] is taken as a 68 % confidence interval. A complication arises, however, when the *p* value from the ANOVA *F* test is greater than *α*/2; by definition, the *p* value is computed under the hypothesis that there is no effect, that is *ω*^2^ = 0. Values of *ω*^2^ cannot be any lower than 0, and hence there are no *ω*^2^ values that would be rejected by the upper tailed test. In this case the lower bound on the CI does not exist. A second complication arises when the *p* value is greater than 1−*α*/2: all lower-tailed tests will reject, and hence the upper bound of the CI does not exist. If a bound does not exist, Steiger (2004) arbitrarily sets it at 0.

To see how this CI works in practice, suppose we design a three-group, between-subjects experiment with *N* = 10 participants in each group and obtain an *F*(2,27)=0.18, *p* = 0.84. Following recommendations for good analysis practices (e.g., Psychonomics society 2012; Wilkinson & the Task Force on Statistical Inference, 1999, we would like to compute a confidence interval on the standardized effects size *ω*^2^. Using software to compute Steiger’s CI, we obtain the 68 % confidence interval [0,0.01].

**Fig. 7:**
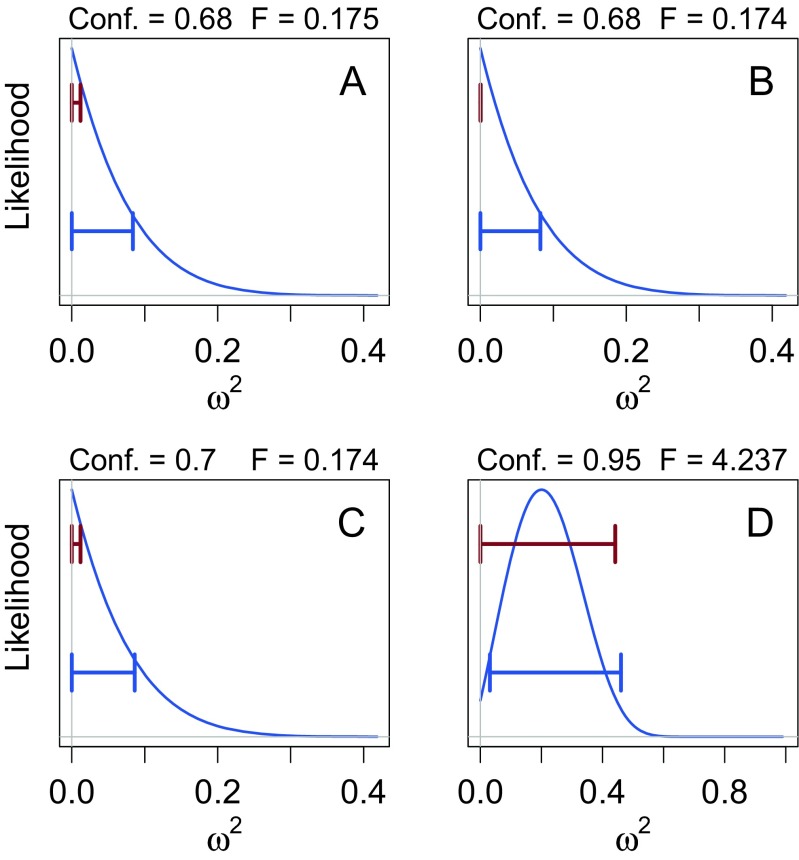
Likelihoods, confidence intervals, and Bayesian credible intervals (highest posterior density, or HPD, intervals) for four hypothetical experimental results. In each figure, the top interval is Steiger’s (2004) confidence interval for *ω* ^2^; the bottom interval is the Bayesian HPD. See text for details

Figure 7a (top interval) shows the resulting 68 % interval. If we were not aware of the fallacies of confidence intervals, we might publish this confidence interval thinking it provides a good measure of the precision of the estimate of *ω*^2^. Note that the lower limit of the confidence interval is exactly 0, because the lower bound did not exist. In discussing this situation Steiger and Fouladi (1997) say

“[Arbitrarily setting the confidence limit at 0] maintains the correct coverage probability for the confidence interval, but the width of the confidence interval may be suspect as an index of the precision of measurement when either or both ends of the confidence interval are at 0. In such cases, one might consider obtaining alternative indications of precision of measurement, such as an estimate of the standard error of the statistic.” (Steiger and Fouladi, 1997, p. 255)

Steiger (2004) further notes that “relationship [between CI width and precision] is less than perfect and is seriously compromised in some situations for several reasons” (p. 177). This is a rather startling admission: a major part of the justification for confidence intervals, including the one computed here, is that confidence intervals supposedly allow an assessment of the precision with which the parameter is estimated. The confidence interval fails to meet the purpose for which it was advocated in the first place, but Steiger does not indicate why, nor under what conditions the CI will successfully track precision.

We can confirm the need for Steiger’s caution — essentially, a warning about the precision fallacy — by looking at the likelihood, which is the probability density of the observed *F* statistic computed for all possible true values of *ω*^2^. Notice how narrow the confidence interval is compared to the likelihood of *ω*^2^. The likelihood falls much more slowly as *ω*^2^ gets larger than the confidence interval would appear to imply, if we believed the precision fallacy. We can also compare the confidence interval to a 68 % Bayesian credible interval, computed assuming standard “noninformative” priors on the means and the error variance.^7^ The Bayesian credible interval is substantially wider, revealing the imprecision with which *ω*^2^ is estimated.

Figure 7b shows the same case, but for a slightly smaller *F* value. The precision with which *ω*^2^ is estimated has not changed to any substantial degree; yet now the confidence interval contains only the value *ω*^2^ = 0: or, more accurately, the confidence interval is empty because this *F* value would always be rejected by one of the pairs of one-sided tests that led to the construction of the confidence interval. As Steiger points out, a “zero-width confidence interval obviously does not imply that effect size was determined with perfect precision,” (p. 177), nor can it imply that there is a 68 % probability that *ω*^2^ is exactly 0. This can be clearly seen by examining the likelihood and Bayesian credible interval.

Some authors (e.g., Dufour, 1997) interpret empty confidence intervals as indicative of model misfit. In the case of this one sample design, if the confidence interval is empty then the means are more similar than would be expected even under the null hypothesis *α*/2 of the time; that is, *p* > 1−*α*/2, and hence *F* is small. If this model rejection significance test logic is used, the confidence interval itself becomes uninterpretable as the model gets close to rejection, because it appears to indicate false precision (Gelman 2011). Moreover, in this case the *p* value is certainly more informative than the CI; the *p* value provides graded information that does not depend on the arbitrary choice of *α*, while the CI is simply empty for all values of *p* > 1−*α*/2.

Panel C shows what happens when we increase the confidence coefficient slightly to 70 %. Again, the precision with which the parameter is estimated has not changed, yet the confidence interval now again has nonzero width.

Figure 7d shows the results of an analysis with *F*(2,27)=4.24, *p* = 0.03, and using a 95 % confidence interval. Steiger’s interval has now encompassed most of the likelihood, but the lower bound is still “stuck” at 0. In this situation, Steiger and Fouladi advise us that the width of the CI is “suspect” as an indication of precision, and that we should “obtain[] [an] alternative indication[] of precision of measurement.” As it turns out, here the confidence interval is not too different from the credible interval, though the confidence interval is longer and is unbalanced. However, we would not know this if we did not examine the likelihood and the Bayesian credible interval; the only reason we know the confidence interval has a reasonable width in this particular case is its agreement with the actual measures of precision offered by the likelihood and the credible interval.

How often will Steiger’s confidence procedure yield a “suspect” confidence interval? This will occur whenever the *p* value for the corresponding *F* test is *p* > *α*/2; for a 95 % confidence interval, this means that whenever *p* > 0.025, Steiger and Fouladi recommend against using the confidence interval for precisely the purpose that they — and other proponents of confidence intervals — recommend it for. This is not a mere theoretical issue; moderately-sized *p* values often occur. In a cursory review of papers citing Steiger (2004), we found many that obtained and reported, without note, suspect confidence intervals bounded at 0 (e.g., Cumming, Sherar, Gammon, Standage, & Malina, 2012; Gilroy & Pearce 2014; Hamerman & Morewedge, 2015; Lahiri, Maloney, Rogers, & Ge, 2013; Hamerman & Morewedge,^2015^; Todd, Vurbic, & Bouton, 2014; Winter et al., 2014). The others did not use confidence intervals, instead relying on point estimates of effect size and *p* values (e.g., Hollingdale & Greitemeyer, 2014); but from the *p* values it could be inferred that if they had followed “good practice” and computed such confidence intervals, they would have obtained intervals that according to Steiger could not be interpreted as anything but an inverted *F* test.

It makes sense, however, that authors using confidence intervals would not note that the interpretation of their confidence intervals is problematic. If confidence intervals truly contained the most likely values, or if they were indices of the precision, or if the confidence coefficient indexed the uncertainty we should have that the parameter is in an interval, then it would seem that a CI is a CI: what you learn from one is the same as what you learn from another. The idea that the *p* value can determine whether the interpretation of a confidence interval is possible is not intuitive in light of the way CIs are typically presented.

We see no reason why our ability to interpret an interval *should* be compromised simply because we obtained a *p* value that was not low enough. Certainly, the confidence coefficient is arbitrary; if the width is suspect for one confidence coefficient, it makes little sense that the CI width would become acceptable just because we changed the confidence coefficient so the interval bounds did not include 0. Also, if the width is too narrow with moderate *p* values, such that it is not an index of precision, it seems that the interval will be too wide in other circumstances, possibly threatening the interpretation as well. This was evident with the UMP procedure in the submersible example: the UMP interval was too narrow when the data provided little information, and was too wide when the data provided substantial information.

Steiger and Fouladi (1997) summarize the central problem with confidence intervals when they say that in order to maintain the correct coverage probability — a frequentist pre-data concern — they sacrifice the very thing researchers want confidence intervals to be: a post-data index of the precision of measurement. If our goal is to move away from significance testing, we should not use methods which cannot be interpreted except as inversions of significance tests. We agree with Steiger and Fouladi that researchers should consider obtaining alternative indications of precision of measurement; luckily, Bayesian credible intervals fit the bill rather nicely, rendering confidence intervals unnecessary.

## Discussion

Using the theory of confidence intervals and the support of two examples, we have shown that CIs do not have the properties that are often claimed on their behalf. Confidence interval theory was developed to solve a very constrained problem: how can one construct a procedure that produces intervals containing the true parameter a fixed proportion of the time? Claims that confidence intervals yield an index of precision, that the values within them are plausible, and that the confidence coefficient can be read as a measure of certainty that the interval contains the true value, are all fallacies and unjustified by confidence interval theory.

Good intentions underlie the advocacy of confidence intervals: it would be desirable to have procedures with the properties claimed. The FCF is driven by a desire to assess the plausibility that an interval contains the true value; the likelihood fallacy is driven by a desire to determine which values of the parameter should be taken seriously; and the precision fallacy is driven by a desire to quantify the precision of the estimates. We support these goals (Morey et al. 2014), but confidence interval theory is not the way to achieve them.

### Guidelines for interpreting and reporting intervals

Frequentist theory can be counter-intuitive at times; as Fisher was fond of pointing out, frequentist theorists often seemed disconnected with the concerns of scientists, developing methods that did not suit their needs (e.g., Fisher, 1955, p. 70). This has lead to confusion where practitioners assume that methods designed for one purpose were really meant for another. In order to help mitigate such confusion, here we would like to offer readers a clear guide to interpreting and reporting confidence intervals.

Once one has collected data and computed a confidence interval, how does one then interpret the interval? The answer is quite straightforward: one does not – at least not within confidence interval theory.^8^ As Neyman and others pointed out repeatedly, and as we have shown, confidence limits cannot be interpreted as anything besides the result of a procedure that will contain the true value in a fixed proportion of samples. Unless an interpretation of the interval can be specifically justified by some *other* theory of inference, confidence intervals must remain uninterpreted, lest one make arbitrary inferences or inferences that are contradicted by the data. This applies even to “good” confidence intervals, as these are often built by inverting significance tests and may have strange properties (e.g., Steiger, 2004).

In order to help mitigate confusion in the scientific literature, we suggest the following guidelines for reporting of intervals informed by our discussion in this manuscript.

#### Report credible intervals instead of confidence intervals

We believe any author who chooses to use confidence intervals should ensure that the intervals correspond numerically with credible intervals under some reasonable prior. Many confidence intervals cannot be so interpreted, but if the authors know they can be, they should be called “credible intervals”. This signals to readers that they can interpret the interval as they have been (incorrectly) told they can interpret confidence intervals. Of course, the corresponding prior must also be reported. This is not to say that one cannot also refer to credible intervals as confidence intervals, if indeed they are; however, readers are likely more interested in knowing that the procedure allows valid post-data inference — not pre-data inference — if they are interested arriving at substantive conclusions from the computed interval.

#### Do not use confidence procedures whose Bayesian properties are not known

As Casella (1992) pointed out, the post-data properties of a procedure are necessary for understanding what can be inferred from an interval. Any procedure whose Bayesian properties have not been explored may have properties that make it unsuitable for post-data inference. Procedures whose properties have not been adequately studied are inappropriate for general use.

#### Warn readers if the confidence procedure does not correspond to a Bayesian procedure

If it is known that a confidence interval does not correspond to a Bayesian procedure, warn readers that the confidence interval cannot be interpreted as having an *X*% probability of containing the parameter, that cannot be interpreted in terms of the precision of measurement, and that cannot be said to contain the values that should be taken seriously: the interval is merely an interval that, prior to sampling, had an *X*% probability of containing the true value. Authors choosing to report CIs have a responsibility to keep their readers from invalid inferences, because it is almost certain that without a warning readers will misinterpret them (Hoekstra et al. 2014).

#### *Never* report a confidence interval without noting the procedure and the corresponding statistics

As we have described, there are many different ways to construct confidence intervals, and they will have different properties. Some will have better frequentist properties than others; some will correspond to credible intervals, and others will not. It is unfortunately common for authors to report confidence intervals without noting how they were constructed or even citing a source. As can be seen from the examples we have presented, this is a terrible practice: without knowing which confidence procedure was used, it is unclear what can be inferred. In the submersible example, consider a 50 % confidence interval .5 meters wide. This could correspond to very precise information (Bayesian interval) or very imprecise information (UMP and nonparametric interval). Not knowing which procedure was used could lead to absurd inferences. In addition, enough information should be presented so that any reader can compute a different confidence interval or credible interval. In many cases, this is covered by standard reporting practices, but in other cases more information may need to be given.

#### Consider reporting likelihoods or posteriors instead

An interval provides fairly impoverished information. Just as proponents of confidence intervals argue that CIs provide more information than a significance test (although this is debatable for many CIs), a likelihood or a posterior provides much more information than an interval. Recently, Cumming (2014) has proposed so-called “cat’s eye” intervals which correspond to Bayesian posteriors under a “non-informative” prior for normally distributed data. With modern scientific graphics so easy to create, we see no reason why likelihoods and posteriors cannot augment or even replace intervals in most circumstances (e.g., Kruschke, 2010). With a likelihood or a posterior, the arbitrariness of the confidence or credibility coefficient is avoided altogether.

A complete account of Bayesian statistics is beyond the scope of this paper (and indeed, can fill entire courses). In recent years, a number of good resources have been developed for readers wishing to learn more about applied Bayesian statistics, including estimation of posterior distributions and credible intervals: on the less technical side, there are texts by Bolstad (2007), Lee and Wagenmakers (2013), and Lynch (2007); on the more technical side are texts by Jackman (2009),Ntzoufras (2009), and Gelman et al. (2004). There are also numerous resources on the world wide web to help beginners. For readers wishing to try some simple examples, the supplement to this article contains R code to estimate posterior distributions and credible intervals for the examples in this paper.

### Confidence intervals versus credible intervals

One of the misconceptions regarding the relationship between Bayesian inference and frequentist inference is that they will lead to the same inferences, and hence all confidence intervals can simply be interpreted in a Bayesian way. In the case where data are normally distributed, for instance, there is a particular prior that will lead to a confidence interval that is numerically identical to Bayesian credible intervals computed using the Bayesian posterior (Jeffreys 1961; Lindley 1965). This might lead one to suspect that it does not matter whether one uses confidence procedures or Bayesian procedures. We showed, however, that confidence intervals and credible intervals can disagree markedly. The only way to know that a confidence interval is numerically identical to some credible interval is to *prove* it. The correspondence cannot — and should not — be assumed.

More broadly, the defense of confidence procedures by noting that, in some restricted cases, they numerically correspond to Bayesian procedures is actually no defense at all. One must first choose which confidence procedure, of many, to use; if one is committed to the procedure that allows a Bayesian interpretation, then one’s time is much better spent simply applying Bayesian theory. If the benefits of Bayesian theory are desired — and they clearly are, by proponents of confidence intervals — then there is no reason why Bayesian inference should not be applied in its full generality, rather than using the occasional correspondence with credible intervals as a hand-waving defense of confidence intervals.

It is important to emphasize, however, that for many of the confidence procedures presented in the applied statistical literature, no effort has been made to show that the intervals have the properties that proponents of confidence intervals desire. We should expect, as a matter of course, that developers of new confidence intervals show that their intervals have the desired inferential properties, instead of just nominal coverage of the true value and “short” width. Because developers of confidence intervals have not done this, the push for confidence intervals rests on uncertain ground. Adopting Bayesian inference, where all inferences arise within a logical, unified framework, would render the problems of assessing the properties of these confidence procedures moot. If desired, coverage of a Bayesian procedure can also be assessed; but if one is interested primarily in reasonable post-data inference, then Bayesian properties should be the priority, not frequentist coverage (cf. Gelman, 2008; Wasserman, 2008).

For advocates of reasoning by intervals, adopting Bayesian inference would have other benefits. The end-points of a confidence interval are always set by the data. Suppose, however, we are interested in determining the plausibility that a parameter is in a particular range; for instance, in the United States, it is against the law to execute criminals who are intellectually disabled. The criterion used for intellectual disability in the US state of Florida is having a true IQ less than 70. Since IQ is measured with error, one might ask what confidence we have that a particular criminal’s true IQ is less than 70 (see Anastasi and Urbina (1997), or Cronbach (1990), for an overview of confidence intervals for IQ). In this case, the interval we would like to assess for plausibility is no longer a function of the sample. The long-run probability that the true value is inside a fixed interval is unknown and is either 0 or 1, and hence no confidence procedure can be constructed, even though such information may be critically important to a researcher, policy maker, or criminal defendant (Pratt et al. 1995).

Even in seemingly simple cases where a fixed interval is nested inside a CI, or *vice versa*, one cannot draw conclusions about the plausibility of a fixed interval. One might assume that an interval nested within a CI must have lower confidence than the CI, given that it is shorter; however, as shown in Fig. 1b, a 100 % confidence interval (the likelihood) is nested within some of the 50 % confidence intervals. Likewise, one might believe that if a CI is nested within a fixed interval, then the fixed interval must have greater probability than the interval. But in Fig. 1a, one can imagine a fixed interval just larger than the 50 % UMP interval; this will have much lower than 50 % probability of containing the true value, due to the fact that it occupies a small proportion of the likelihood. Knowledge that the FCF is a fallacy prohibits one from using confidence intervals to assess the probability of fixed intervals. Bayesian procedures, on the other hand, offer the ability to compute the plausibility of any given range of values. Because all such inferences must be made from the posterior distribution, inferences must remain mutually consistent (Lindley, 1985; see also Fisher, 1935, for a similar argument).

Moving to credible intervals from confidence intervals would necessitate a shift in thinking, however, away from a test-centric view with respect to intervals (e.g., “is 0 in the interval?”). Although every confidence interval can be interpreted as a test, credible intervals cannot be so interpreted. Assessing the Bayesian credibility of a specific parameter value by checking whether it is included in a credible interval is, as Berger (2006) puts it, “simply wrong.” When testing a specific value is of interest (such as a null hypothesis), that specific value must be assigned non-zero probability *a priori*. While not conceptually difficult, it is beyond the scope of this paper; see Rouder et al. (2009), Wagenmakers et al. (2008), or Dienes (2011) for accessible accounts.

Finally, we believe that in science, the meaning of our inferences are important. Bayesian credible intervals support an interpretation of probability in terms of plausibility, thanks to the explicit use of a prior. Confidence intervals, on the other hand, are based on a philosophy that does not allow inferences about plausibility, and does not utilize prior information. Using confidence intervals as if they were credible intervals is an attempt to smuggle Bayesian meaning into frequentist statistics, without proper consideration of a prior. As they say, there is no such thing as a free lunch; one must choose. We suspect that researchers, given the choice, would rather specify priors and get the benefits that come from Bayesian theory. We should not pretend, however, that the choice need not be made. Confidence interval theory and Bayesian theory are not interchangeable, and should not be treated as so.

### Conclusion

We have suggested that confidence intervals do not support the inferences that their advocates believe they do. It is an interesting question how the theory of confidence intervals began with Neyman as a method of avoiding the problem of reasoning from data by making dichotomous statements (Neyman 1937, 1941), eventually becoming a method that many believe is the best way to reason from data (e.g. Cumming & Finch, 2005; Cumming & Fidler, 2009) and a way to avoid dichotomous statements (e.g. Cumming, 2014; Hoekstra, Finch, Kiers, & Johnson, 2006; Wilkinson & the Task Force on Statistical Inference, 1999). Regardless of how this confusion started, we believe it should be recognized that confidence interval theory offers only the shallowest of interpretations, and is not well-suited to the needs of scientists.

We do not believe that the theory of confidence intervals provides a viable foundation for the future of psychological methods. Confidence procedures that do not have Bayesian properties have other undesirable properties; confidence procedures that *do* have Bayesian properties can be justified using Bayesian theory. If we were to give up the use of confidence procedures, what would we lose? Abandoning the use of confidence procedures means abandoning a method that merely allows us to create intervals that include the true value with a fixed long-run probability. We suspect that if researchers understand that this is the only thing they will be losing, they will not consider it a great loss. By adopting Bayesian inference, they will gain a way of making principled statements about precision and plausibility. Ultimately, this is exactly what the advocates of CIs have wanted all along.

## Electronic supplementary material

(PDF 206 KB)

## Footnotes

1 The modern definition of a confidence interval allows the probability to be at least X%, rather than exactly X%. This detail does not affect any of the points we will make; we mention it for completeness.

2 Neyman first articulated the theory in another paper before his major theoretical paper in 1937.

3 John Tukey has said that the collective noun for a group of statisticians is “quarrel” (McGrayne 2011).

4 In order to construct a better interval, a frequentist would typically truncate the interval to only the possible values, as was done in generating the UMP procedure from the nonparametric procedure (e.g., Spanos, 2011). This is guaranteed to lead to a better procedure. Our point here is that it is a mistake to naively assume that a procedure has good properties on the basis that it is a confidence procedure. However, see Velicer et al. (2008) for an example of CI proponents including impossible values in confidence intervals, and Fidler and Thompson (2001) for a defense of this practice.

5 Several readers of a previous draft of this manuscript noted that frequentists use the likelihood as well, and so may prefer the Bayesian procedure in this example. However, as Neyman (1977) points out, the likelihood has no special status to a frequentist; what matters is the frequentist properties of the procedure, not how it was constructed.

6 This should not be taken to mean that inference by confidence intervals is not problematic even in this simple case; see e.g., Brown (1967) and Buehler & Feddersen (1963).

7 See the supplement for details. We do not generally advocate non-informative priors on parameters of interest (Rouder et al. 2012; Wetzels et al. 2012); in this instance we use them as a comparison because many people believe, incorrectly, that confidence intervals numerically correspond to Bayesian credible intervals with noninformative priors.

8 Some recent writers have suggested the replacement of Neyman’s behavioral view on confidence intervals with a frequentist view focused on tests at various levels of “stringency” (see, e.g., Mayo & Cox, 2006; Mayo & Spanos, 2006). Readers who prefer a frequentist paradigm may wish to explore this approach; however, we are unaware of any comprehensive account of CIs within this paradigm, and regardless, it does not offer the properties desired by CI proponents. This is not to be read as an argument against it, but rather a warning that one must make a choice.
