# Supplementary material for: The fallacy of placing confidence in confidence intervals
Source: Psychon Bull Rev. 2015 Oct 8;23:103–23. doi: 10.3758/s13423-015-0947-8 (PMC4742505; doi:10.3758/s13423-015-0947-8)
Supplement: Supplementary file 1 — (PDF 206 KB) [file 13423_2015_947_MOESM1_ESM.pdf]

Supplement to "The Fallacy of Placing Confidence in Confidence Intervals"

Richard D. Morey (richarddmorey@gmail.com),
Rink Hoekstra, Jeffrey N. Rouder,
Michael D. Lee, Eric-Jan Wagenmakers

July 30, 2015

The source code for this document, and the main manuscript, is available at https://github.com/richarddmorey/ConfidenceIntervalsFallacy.

1 The lost submarine: details

We presented a situation where $N = 2$ observations were distributed uniformly:

$$y_i \overset{iid}{\sim} \text{Uniform}(\theta - 5, \theta + 5),\ i = 1, \ldots, N$$

and the goal is to estimate θ, the location of the submarine hatch. Without loss of generality we denote x_1 as the smaller of the two observations. In the text, we considered five 50% confidence procedures; in this section, we give the details about the sampling distribution procedure and the Bayes procedure that were omitted from the text.

1.1 Sampling distribution procedure

Consider the sample mean, $\bar{y} = (y_1 + y_2)/2$. As the sum of two uniform deviates, it is a well-known fact that $\bar{y}$ will have a triangular distribution with location θ and minimum and maximum $\theta - 5$ and $\theta + 5$, respectively. This distribution is shown in Figure 1.

It is desired to find the width of the base of the shaded region in Figure 1 such that it has an area of .5. To do this we first find the width of the base of the unshaded triangular area marked "a" in Figure 1 such that the area of the triangle is .25. The corresponding unshaded triangle on the left side will also have area .25, which means that since the figure is a density, the shaded region must have the remaining area of .5. Elementary geometry will show that the width of the base of triangle "a" is $5/\sqrt{2}$, meaning that the distance between θ and the altitude of triangle "a" is $5 - 5/\sqrt{2}$ or about 1.46m.

We can thus say that

$$Pr(-(5 - 5/\sqrt{2}) < \bar{y} - \theta < 5 - 5/\sqrt{2}) = .5$$

which implies that, in repeated sampling,

$$Pr(\bar{y} - (5 - 5/\sqrt{2}) < \theta < \bar{y} + (5 - 5/\sqrt{2})) = .5$$

which defines the sampling distribution confidence procedure. This is an example of using $\bar{y} - \theta$ as a pivotal quantity (Casella & Berger, 2002).

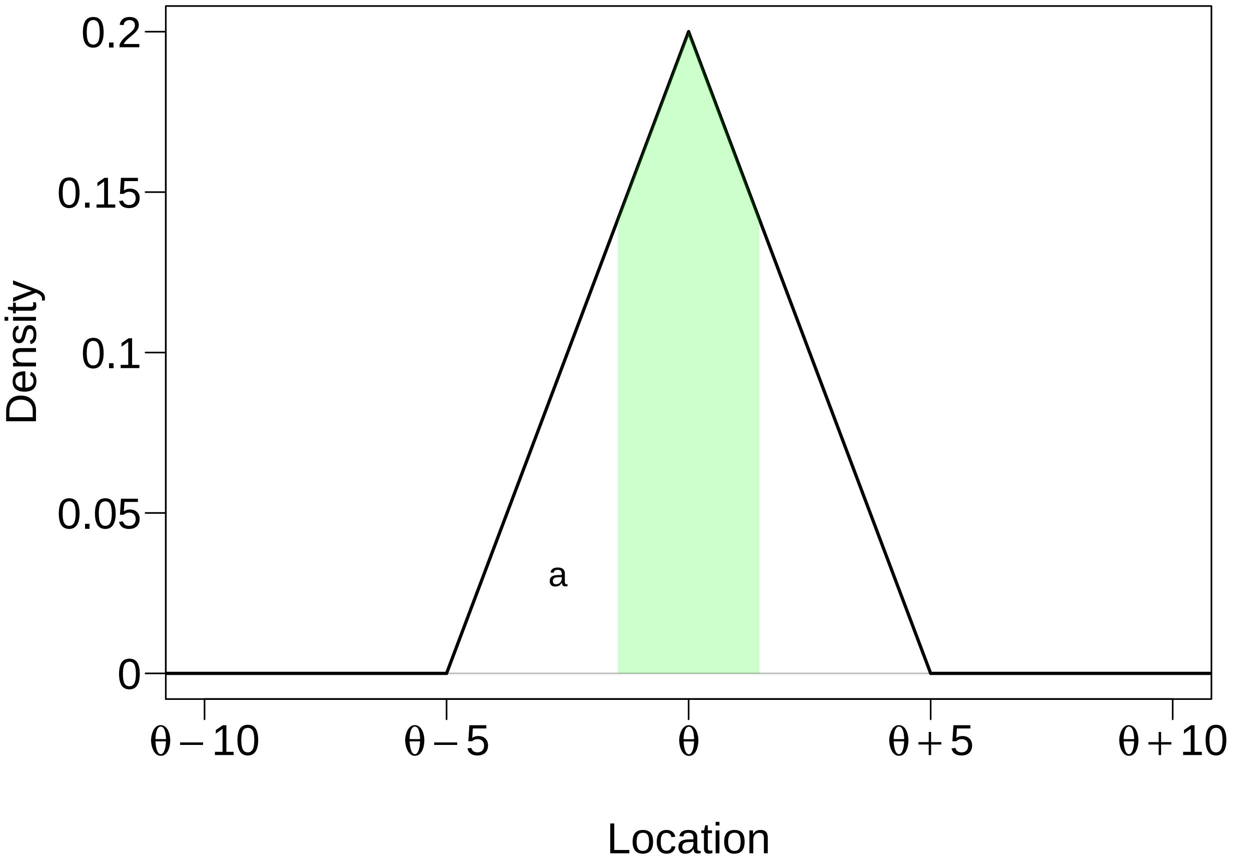

Figure 1: The sampling distribution of the mean $\bar{x}$ in the submarine scenario. The shaded region represents the central 50% of the area. The unshaded triangle marked "a" has area .25, and the standard deviation of this sampling distribution is about 2.04.

We can also derive the standard deviation of the sampling distribution of $\bar{y}$, also called the standard error. It is defined as:

$$SE(\bar{y}) = \sqrt{V(\bar{y})} = \sqrt{\int_{-5}^{5} z^2 p(z)\, dz}$$

where $p(z)$ is the triangular sampling distribution in Figure 1 centered around $\theta = 0$. Solving the integral yields

$$SE(\bar{y}) = \frac{5}{\sqrt{6}} \approx 2.04.$$

1.2 Bayesian procedure

The posterior distribution is proportional to the likelihood times the prior. The likelihood is

$$p(y_1, y_2 \mid \theta) \propto \prod_{i=1}^{2} \mathcal{I}(\theta - 5 < y_i < \theta + 5);$$

where $\mathcal{I}$ is an indicator function. Note since this is the product of two indicator functions, it can only be nonzero when both indicator functions' conditions are met; that is, when $y_1 + 5$ and $y_2 + 5$ are both greater than θ, and $y_1 - 5$ and $y_2 - 5$ are both less than θ. If the minimum of $y_1 + 5$ and $y_2 + 5$ is greater than θ, then so to must be the maximum. The likelihood thus can be rewritten

$$p(x_1, x_2 \mid \theta) \propto \mathcal{I}(x_2 - 5 < \theta < x_1 + 5);$$

where x_1 and x_2 are the minimum and maximum observations, respectively. If the prior for θ is proportional to a constant, then the posterior is

$$p(\theta \mid x_1, x_2) \propto \mathcal{I}(x_2 - 5 < \theta < x_1 + 5),$$

This posterior is a uniform distribution over all *a posteriori* possible values of θ (that is, all θ values within 5 meters of all observations), has width

$$10 - (x_2 - x_1),$$

and is centered around $\bar{x}$. Because the posterior comprises all values of θ the data have not ruled out – and is essentially just the classical likelihood – the width of this posterior can be taken as an indicator of the precision of the estimate of θ.

The middle 50% of the likelihood can be taken as a 50% objective Bayesian credible interval. Proof that this Bayesian procedure is also a confidence procedure is trivial and can be found in Welch (1939).

1.3 BUGS implementation

The submersible example was selected in part because it is so trivial; the confidence intervals and Bayesian credible intervals can be derived with very little effort. However, for more complicated problems, credible intervals can be more challenging to derive. Thankfully, modern Bayesian software tools make estimation of credible intervals in many problems as trivial as stating the problem along with priors on the parameters.

BUGS is a special language that allows users to define a model and prior. Using a software that interprets the BUGS language, such as JAGS (Plummer, 2003) or WinBUGS (Lunn, Thomas, Best, & Spiegelhalter, 2000), the model and prior are then combined with the data. The software then outputs samples from the posterior distribution for all the parameters, which can be used to create credible intervals.

A full explanation of how to use the BUGS language is beyond the scope of this supplement. Readers can find more information about using BUGS in Ntzoufras (2009), Lee and Wagenmakers (2013), and

many tutorials are available on the world wide web. Here, we show how to obtain a credible interval for the submersible Example 1 using JAGS; in a later section we show how to obtain a confidence interval for Example 2, ω^2 in ANOVA designs.

We first define the model and prior in R using the BUGS language. Notice that this is simply stating the distributions of the data points, along with a prior for θ.

```
BUGS_model = "
  model{
  y1 ~ dunif(theta - 5, theta + 5)
  y2 ~ dunif(theta - 5, theta + 5)
  theta ~ dnorm( theta_mean, theta_precision)
  }
"
```

We now define a list of values that will get passed to JAGS. `y1` and `y2` are the data observed values from Figure 1A, and the prior we choose is an informative prior for demonstration.

```
for_JAGS = list( y1 = -4.5,
                 y2 = 4.5,
                 theta_mean = -2.5,
                 theta_precision = 1/10^2 )
```

Since precision is the reciprocal of variance, the prior on θ corresponds to a Normal$(\mu = -2.5, \sigma = 10)$ prior. All that remains is to load JAGS and combine the model information in `BUGS_model` with the data in `for_JAGS`, then to obtain samples from the posterior distribution.

```
# Load the rjags package to interface with JAGS
require( rjags )

## Loading required package:  rjags
## Loading required package:  coda
## Linked to JAGS 3.4.0
## Loaded modules:  basemod,bugs

# Set initial value for the sampler
initial.values = list(theta = 0)

# Combine the model with the data
compiled_model = jags.model( file = textConnection(BUGS_model),
                             data = for_JAGS, inits = initial.values,
                             quiet = TRUE )

# Sample from the posterior distribution
posterior_samples = coda.samples( model = compiled_model,
                                  variable.names = c("theta"),
                                  n.iter = 100000 )
```

We can now plot the samples we obtained using the `hist` function in R.

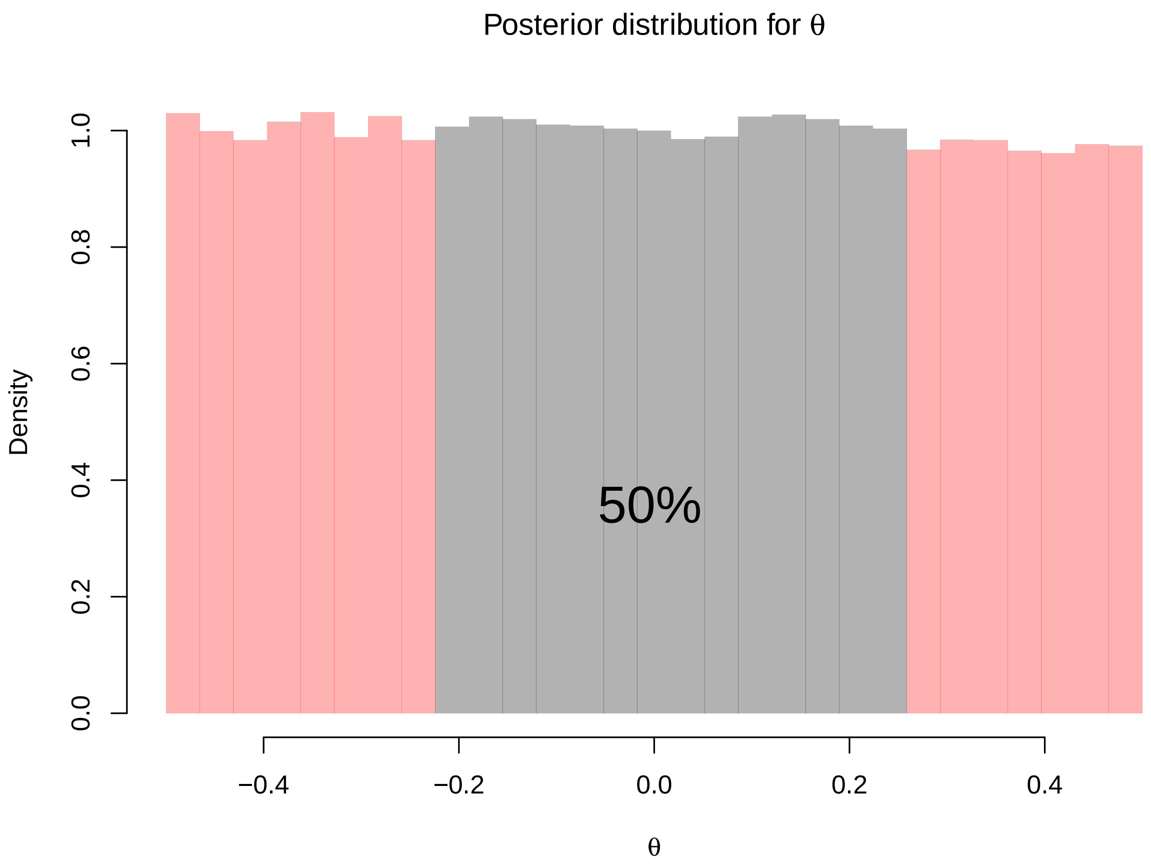

Figure 2: Posterior distribution for θ, estimated using JAGS.

```
theta_samples = posterior_samples[[ 1 ]][ , "theta" ]
hist( theta_samples, breaks = 20, freq = FALSE )
```

Note the resemblance to Figure 5, bottom panel, in the manuscript. We use the `summary` function on the samples to obtain a point estimate as well as quantiles of the posterior distribution, which can be used to form credible intervals. The 50% central credible interval is the interval between the 25th and 75th percentile.

```
summary(theta_samples)

##
## Iterations = 1001:101000
## Thinning interval = 1
## Number of chains = 1
## Sample size per chain = 1e+05
##
## 1. Empirical mean and standard deviation for each variable,
##    plus standard error of the mean:
```

```
##
##            Mean             SD       Naive SE Time-series SE
##      -0.0032434      0.2877994      0.0009101      0.0010831
##
## 2. Quantiles for each variable:
##
##      2.5%       25%       50%       75%     97.5%
## -0.475826 -0.252609 -0.004236  0.243511  0.474247
```

2 Credible interval for ω^2: details

In the manuscript, we compare Steiger's (2004) confidence intervals for ω^2 to Bayesian highest posterior density (HPD) credible intervals. In this section we describe how the Bayesian HPD intervals were computed.

Consider a one-way design with J groups and N observations in each group. Let y_{ij} be the ith observation in the jth group. Also suppose that

$$y_{ij} \overset{indep.}{\sim} \text{Normal}(\mu_j, \sigma^2)$$

where μ_j is the population mean of the jth group and σ^2 is the error variance. We assume a "non-informative" prior on parameters $\boldsymbol{\mu}, \sigma^2$:

$$p(\mu_1, \ldots, \mu_J, \sigma^2) \propto (\sigma^2)^{-1}.$$

This prior is flat on $(\mu_1, \ldots, \mu_J, \log \sigma^2)$. In application, it would be wiser to assume an informative prior on these parameters, in particular assuming a population over the μ parameters or even the possibility that $\mu_1 = \ldots = \mu_J = 0$ (Rouder, Morey, Speckman, & Province, 2012). However, for this manuscript we compare against a "non-informative" prior in order to show the differences between the confidence interval and the Bayesian result with "objective" priors.

Assuming the prior above, an elementary Bayesian calculation (Gelman, Carlin, Stern, & Rubin, 2004) reveals that

$$\sigma^2 \mid \boldsymbol{y} \sim \text{Inverse Gamma}(J(N-1)/2, S/2)$$

where S is the error sum-of-squares from the corresponding one-way ANOVA, and

$$\mu_j \mid \sigma^2, \boldsymbol{y} \overset{indep.}{\sim} \text{Normal}(\bar{x}_j, \sigma^2/N)$$

where μ_j and $\bar{x}_j$ are the true and observed means for the jth group. Following Steiger (2004) we can define

$$\alpha_j = \mu_j - \frac{1}{J}\sum_{j=1}^{J} \mu_j$$

as the deviation from the grand mean of the jth group, and

$$\begin{aligned} \lambda &= N \sum_{j=1}^{J} \left(\frac{\alpha}{\sigma}\right)^2 \\ \omega^2 &= \frac{\lambda}{\lambda + NJ}. \end{aligned}$$

It is then straightforward to set up an MCMC sampler for ω^2. Let M be the number of MCMC iterations desired. We first sample M samples from the marginal posterior distribution of σ^2, then sample the group means from the conditional posterior distribution for $\mu_1, \ldots, \mu_J$. Using these posterior samples, M posterior samples for λ and ω^2 can be computed.

The following R function will sample from the marginal posterior distribution of ω^2:

```
## Assumes that data.frame y has two columns:
## $y is the dependent variable
## $grp is the grouping variable, as a factor
Bayes.posterior.omega2

## function (y, conf.level = 0.95, iterations = 10000)
## {
##     J = nlevels(y$grp)
##     N = nrow(y)/J
##     aov.results = summary(aov(y ~ grp, data = y))
##     SSE = aov.results[[1]][2, 2]
##     sig2 = 1/rgamma(iterations, J * (N - 1)/2, SSE/2)
##     lambda = matrix(NA, iterations)
##     group.means = tapply(y$y, y$grp, mean)
##     for (m in 1:iterations) {
##         mu = rnorm(J, group.means, sqrt(sig2[m]/N))
##         lambda[m] = N * sum((mu - mean(mu))^2/sig2[m])
##     }
##     mcmc(lambda/(lambda + N * J))
## }
```

The `Bayes.posterior.omega2` function can be used to compute the posterior and HPD for the first example in the manuscript. The `fake.data.F` function, defined in the file `steiger.utility.R` (available with the manuscript source code at https://github.com/richarddmorey/ConfidenceIntervalsFallacy), generates a data set with a specified F statistic.

```
cl = .683 ## Confidence level corresponding to standard error
J = 3 ## Number of groups
N = 10 ## observations in a group

df1 = J - 1
df2 = J * (N - 1)

## F statistic from manuscript
Fstat = 0.1748638

set.seed(1)
y = fake.data.F(Fstat, df1, df2)

## Steiger confidence interval
steigerCI = steigerCI.omega2(Fstat,df1,df2, conf.level=cl)
samples.omega2 = Bayes.posterior.omega2(y, cl, 100000)
```

We can compute the Bayesian HPD interval with the `HPDinterval` function in the R package `coda`:

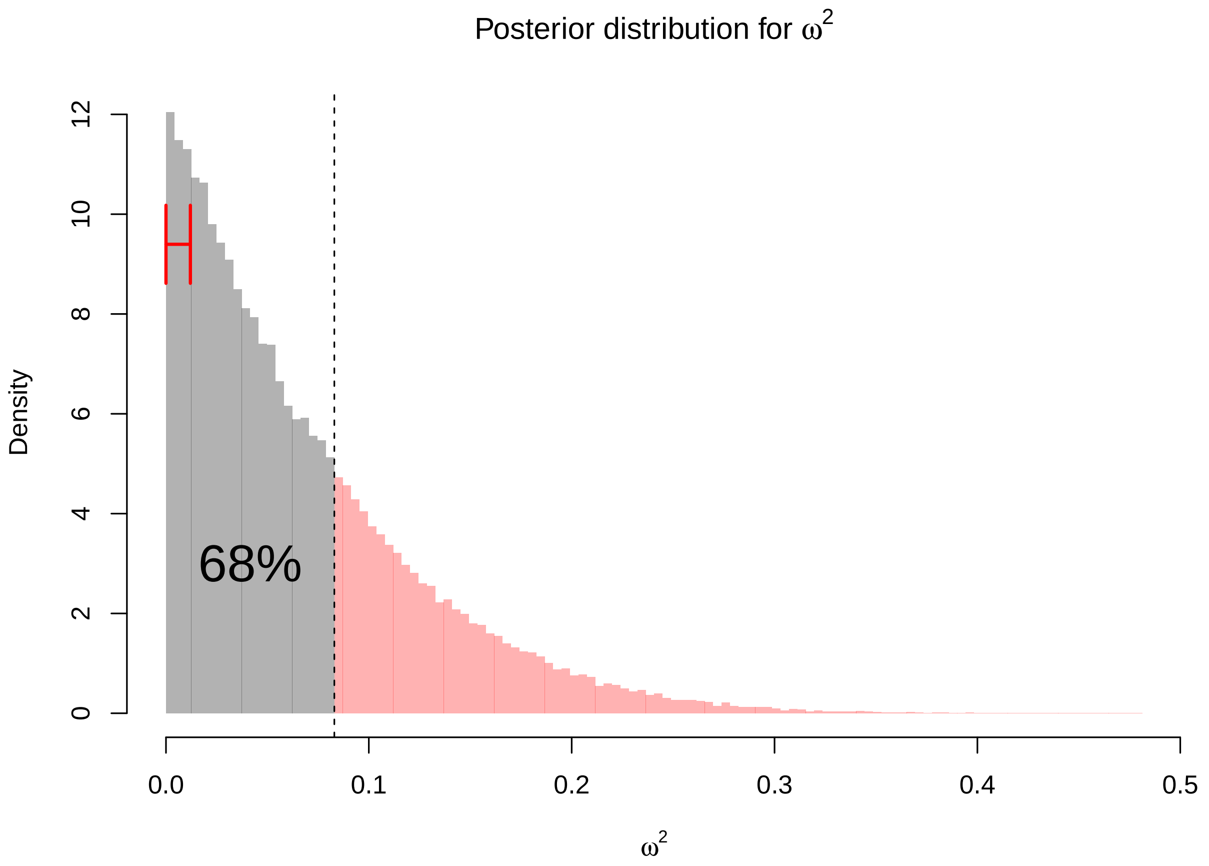

Figure 3: Histogram of the posterior MCMC samples for ω^2. The 68% Bayesian HPD credible interval is highest density region than captures 68% of the posterior density, shown in gray. The vertical dashed line denotes the upper bound of the HPD. The 68% Steiger confidence interval is shown as the interval near the top.

```
library(coda)

HPDinterval( samples.omega2, prob = cl )

##             lower      upper
## var1 5.219606e-06 0.08299102
## attr(,"Probability")
## [1] 0.683
```

2.1 BUGS implementation

Although the code above can be used to quickly sample ω^2 for any one-way design, it is not particularly generalizable for typical users. We can use the BUGS language for Bayesian modeling to create credible intervals in a way that is more accessible to the general user.

```
BUGS_model = "
  model{
    for( i in 1:NJ ){ # iterate over participants
      # Error model for this observation
      y[ i ] ~ dnorm( mu[ group[i] ], precision )
    }

    for( j in 1:J ){ # iterate over groups
      # prior for group mean
      mu[ j ] ~ dnorm( mean_mu, precision_mu )
      # group mean's standardized squared deviation
      # from overall mean
      mu_dev_sq[ j ] <- pow( mu[ j ] - mean( mu ), 2 ) / variance
    }

    # BUGS uses the inverse of the variance (precision)
    # instead of the variance
    precision <- 1 / variance
    # prior on error variance
    variance ~ dgamma( a_variance, b_variance )

    # Define our quantities of interest
    lambda <- N * sum( mu_dev_sq )
    omega2 <- lambda / ( lambda + N * J )
  }
"
```

In the R code below, we define all the constants and the data needed for the analysis, including the prior parameters. These prior parameters were chosen to approximate the "non-informative" prior we used in the previous analysis. As we mentioned in the manuscript, we do not generally advise the use of such non-informative priors; these values are merely chosen for demonstration. In practice, reasonable values would be chosen to inform the analysis.

```
for_JAGS = list( y = y$y,
                 group = y$grp,
                 N = N,
                 J = J,
                 NJ = N*J,
                 mean_mu = 0,
                 precision_mu = 1e-6,
                 a_variance = 1e-6,
                 b_variance = 1e-6 )
```

The following code joins the model (`BUGS_model`) with the data and defined constants (`for_JAGS`) and 10,000 samples from the posterior distribution, outputting the samples of `omega2`, the parameter of interest.

```
# Load the rjags package to interface with JAGS
require( rjags )
```

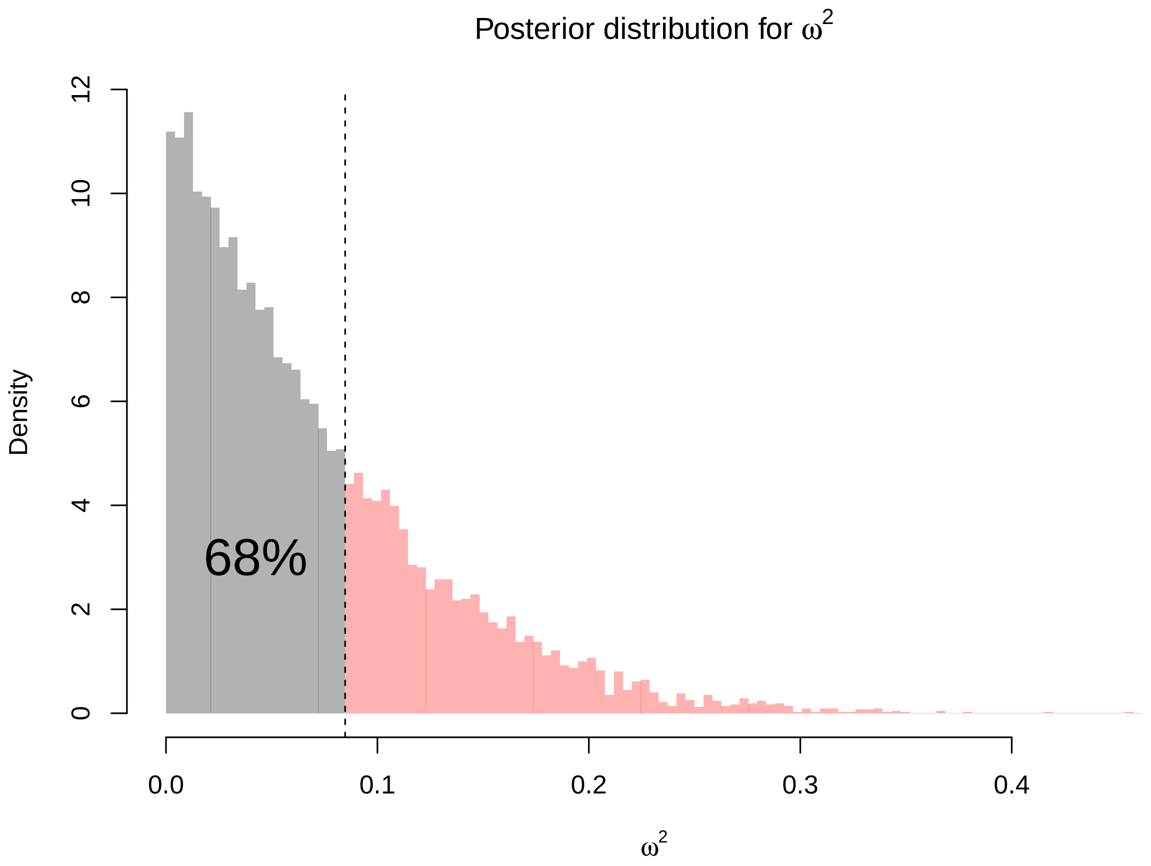

Figure 4: Posterior distribution for ω^2, estimated using JAGS.

```
# Combine the model with the data
compiled_model = jags.model( file = textConnection(BUGS_model),
                             data = for_JAGS, quiet = TRUE )

# Sample from the posterior distribution
posterior_samples = coda.samples( model = compiled_model,
                                  variable.names = c("omega2"),
                                  n.iter = 10000 )
```

The object `posterior_samples` now contains all posterior samples of the parameter ω^2. We can plot their histogram:

```
omega2_samples = posterior_samples[[ 1 ]][ , "omega2" ]
hist( omega2_samples, breaks = 20, freq = FALSE )
```

Note the close similarity between Figure 4 and Figure 3. We can do whatever we like with these samples; of particular interest would be a point estimate and credible interval. For the point estimate, we might select the posterior mean; for the credible interval, we can compute a highest-density region:

```r
# Compute the posterior mean
mean( omega2_samples )
```

```
## [1] 0.06931032
```

```r
# Compute the HDR
HPDinterval( omega2_samples , prob = cl )
```

```
##             lower      upper
## var1 0.0001095948 0.08473403
## attr(,"Probability")
## [1] 0.683
```

Further useful information about the posterior can be obtained using the `summary` function.

```r
summary( omega2_samples )
```

```
##
## Iterations = 1001:11000
## Thinning interval = 1
## Number of chains = 1
## Sample size per chain = 10000
##
## 1. Empirical mean and standard deviation for each variable,
##    plus standard error of the mean:
##
##           Mean             SD       Naive SE Time-series SE
##      0.0693103      0.0593444      0.0005934      0.0005934
##
## 2. Quantiles for each variable:
##
##     2.5%      25%      50%      75%    97.5%
## 0.002287 0.023369 0.053747 0.099825 0.218027
```

References

Casella, G. & Berger, R. L. (2002). *Statistical inference*. Pacific Grove, CA: Duxbury.

Gelman, A., Carlin, J. B., Stern, H. S., & Rubin, D. B. (2004). *Bayesian data analysis (2nd edition)*. London: Chapman and Hall.

Lee, M. D. & Wagenmakers, E.-J. (2013). *Bayesian modeling for cognitive science: a practical course*. Cambridge University Press.

Lunn, D., Thomas, A., Best, N., & Spiegelhalter, D. (2000). WinBUGS – a Bayesian modelling framework: concepts, structure, and extensibility. *Statistics and Computing*, *10*, 325–337.

Ntzoufras, I. (2009). *Bayesian Modeling Using WinBUGS*. Hoboken, New Jersey: Wiley.

Plummer, M. (2003). JAGS: a program for analysis of Bayesian graphical models using Gibbs sampling. In *Proceedings of the 3rd international workshop on distributed statistical computing*.

Rouder, J. N., Morey, R. D., Speckman, P. L., & Province, J. M. (2012). Default Bayes factors for ANOVA designs. *Journal of Mathematical Psychology*, *56*, 356–374. Retrieved from http://dx.doi.org/10.1016/j.jmp.2012.08.001

Steiger, J. H. (2004). Beyond the F test: Effect size confidence intervals and tests of close fit in the analysis of variance and contrast analysis. *Psychological Methods*, *9*(2), 164–182.

Welch, B. L. (1939). On confidence limits and sufficiency, with particular reference to parameters of location. *The Annals of Mathematical Statistics*, *10*(1), 58–69.
